# Systematic Analysis of Gene Expression Alterations and Clinical Outcomes for Long-Chain Acyl-Coenzyme A Synthetase Family in Cancer

**DOI:** 10.1371/journal.pone.0155660

**Published:** 2016-05-12

**Authors:** Wei-Ching Chen, Chih-Yang Wang, Yu-Hsuan Hung, Tzu-Yang Weng, Meng-Chi Yen, Ming-Derg Lai

**Affiliations:** 1 Department of Biochemistry and Molecular Biology, College of Medicine, National Cheng Kung University, Tainan, Taiwan, R.O.C; 2 Institute of Basic Medical Sciences, College of Medicine, National Cheng Kung University, Tainan, Taiwan, R.O.C; 3 Graduate Institute of Clinical Medicine, College of Medicine, Kaohsiung Medical University, Kaohsiung, Taiwan, R.O.C; 4 Center for Infectious Diseases and Signaling Research, College of Medicine, National Cheng Kung University, Tainan, Taiwan, R.O.C; Mayo Clinic College of Medicine, UNITED STATES

## Abstract

Dysregulated lipid metabolism contributes to cancer progression. Our previous study indicates that long-chain fatty acyl-Co A synthetase (ACSL) 3 is essential for lipid upregulation induced by endoplasmic reticulum stress. In this report, we aimed to identify the role of ACSL family in cancer with systematic analysis and *in vitro* experiment. We explored the ACSL expression using Oncomine database to determine the gene alteration during carcinogenesis and identified the association between ACSL expression and the survival of cancer patient using PrognoScan database. ACSL1 may play a potential oncogenic role in colorectal and breast cancer and play a potential tumor suppressor role in lung cancer. Co-expression analysis revealed that ACSL1 was coexpressed with MYBPH, PTPRE, PFKFB3, SOCS3 in colon cancer and with LRRFIP1, TSC22D1 in lung cancer. In accordance with PrognoScan analysis, downregulation of ACSL1 in colon and breast cancer cell line inhibited proliferation, migration, and anchorage-independent growth. In contrast, increase of oncogenic property was observed in lung cancer cell line by attenuating ACSL1. High ACSL3 expression predicted a better prognosis in ovarian cancer; in contrast, high ACSL3 predicted a worse prognosis in melanoma. ACSL3 was coexpressed with SNUPN, TRIP13, and SEMA5A in melanoma. High expression of ACSL4 predicted a worse prognosis in colorectal cancer, but predicted better prognosis in breast, brain and lung cancer. ACSL4 was coexpressed with SERPIN2, HNRNPCL1, ITIH2, PROCR, LRRFIP1. High expression of ACSL5 predicted good prognosis in breast, ovarian, and lung cancers. ACSL5 was coexpressed with TMEM140, TAPBPL, BIRC3, PTPRE, and SERPINB1. Low ACSL6 predicted a worse prognosis in acute myeloid leukemia. ACSL6 was coexpressed with SOX6 and DARC. Altogether, different members of ACSLs are implicated in diverse types of cancer development. ACSL-coexpressed molecules may be used to further investigate the role of ACSL family in individual type of cancers.

## Introduction

Cancer is a leading cause of death worldwide. Many physiological conditions, such as hypoxia, reactive oxygen species (ROS) and metabolic dysfunction, contribute to the cancer progression [1–3]. Fatty acids are important fuel for cell growth and are essential components of cell membranes. Altered fatty acid has been observed in varieties of cancers and is recognized as a marker of cancer. Cancer cell with altered lipid metabolism exhibits the increase of proliferation, progression and metastasis [4]. The elevated de novo fatty acid synthesis is used to meet the energy demand and sustains additional cellular process. Overexpression of fatty acid synthase (FASN) in breast and prostate cancer is associated with the poor prognosis and inhibition of FASN attenuates the lipogenesis and serves as the therapeutic approach [5]. Transcriptomic analysis of metabolite reveals the expression pattern of lipid-associated gene in cancers [6,7].

Fatty acid synthesis starts with the carboxylation of acetyl-CoA to malonyl-CoA via acetyl-CoA carboxylase. The formation of malonyl-CoA provides the 2-carbon donor for fatty acid chain synthesis. Free fatty acid is converted to fatty acyl-CoA by acyl coenzyme A synthetase in an ATP-dependent manner and the unit of fatty acyl-CoA leads to multiple physiological responses and metabolic processes, such as membrane phospholipid biosynthesis, energy usage and storage, and signal lipids [8]. There are five members of acyl coenzyme A synthetase family, including short-chain acyl-CoA synthetases, medium-chain acyl-CoA synthetases, long-chain acyl-CoA synthetases (ACSL), bubblegum acyl-CoA synthetases, and very long-chain acyl-CoA synthetases. Each of the members has unique substrate preference and enzyme activity in different cellular locations. The long-chain acyl-CoA synthetase (ACSL) prefers to the specific substrate of fatty acid with the chain lengths of 12 to 20 carbon atoms [9]. The five isoforms of ACSLs in mammalian are identified as the ACSL1, 3, 4, 5, and 6 [10]. The ACSL1 is abundantly expressed in lipid droplet, microsome and mitochondria and responsible for the elevated levels of the unsaturated fatty acids oleate and linoleate [11]. ACSL3 is present in brain and shows preference for myristate, arachidonate and eicosapentaenoate. ACSL4 favorably utilizes arachidonate as substrate. ACSL5 has a marked preference for palmitic acid, palmitoleic acid, oleic acid and linoleic acid. ACSL6 is found in plasma membrane and displays a high activity with fatty acid with C16-C20 saturated and polyunsaturated [12]. ACSL is the response gene for PPARγ which mediates the lipid metabolism and regulates the caloric absorption [13]. Oncostatin-mediated reduction of plasma LDL-C and total cholesterol is regulated by ACSL3 and ACSL5 [14]. In our previous study, ACSL3 is involved in the endoplasmic reticulum stress-induced lipid accumulation via GSK-3-β. The inhibition of ACSL3 by ACSL inhibitor or GSK-3-β inhibitor reduces the lipid accumulation in liver cell [15]. The increased ACSL3 in U87 human glioblastoma cell drives the tumorigenesis, whereas ACSL3 knockdown reduces lung cancer cell growth [16,17]. In contrast, ACSL3 is decreased in high-grade and metastatic prostate cancer [18]. ACSLs may function in diverse roles in different cancers, indicating the importance of having a comprehensive analysis of ACSL in cancers. Nevertheless, there is no holistic investigation to explore ACSL expression in various types of cancer.

Several databases assessing the gene expression signature of clinical cancer tissue by microarray analysis were established. Oncomine platform includes more than 700 independent datasets with nearly 90,000 microarray experiments. The database identifies the gene expression signature in nearly every pathology-based subtype of cancers [19,20]. The differential expression pattern implies the potential oncogenic role or tumor suppressor role in cancer and leads to a reliable hypothesis for cancer research [21]. We have previously performed a meta-analysis on public microarray datasets and demonstrate voltage-gated calcium gene signatures in clinical cancer patients [22]. Therefore, we aimed to systematically analyze the ACSL expression and identify the survival of cancer patients with high or low ACSL expression from the Oncomine and PrognoScan database, respectively. The co-expression analysis reveals the biological function and provides the information for the possible underlying mechanism. The gene ontology enrichment is performed to predict the gene function and regulation pattern [23]. In this report, we identified the co-expression profiles of each ACSL isoform from the Oncomine database and performed GeneGo Metacore analysis to unveil the biological function. The analyses demonstrate the significance of each ACSL isoform in tumor formation of various kinds of cancer and the association of expression level with survival of cancer patient. Furthermore, the *in vitro* data supports the experimental evidence for a set of accurate prediction from online database, having a better understanding of ACSL in cancers. To our best knowledge, this is the first systematic analysis indicating the role of ACSL family in cancer progression.

## Material and Method

### Oncomine database analysis

The expression of ACSL family members in various types of cancers was identified from Oncomine database using the analysis of “Gene summary view” and “Dataset view” (https://www.oncomine.org/resource/login.html) [19,20]. The mRNA expression fold in cancer tissue compared to the normal tissue was obtained as the parameters of p-value<1E-4, fold change>2, and gene ranking in the top 10% and the analyses were summarized in S1, S3, S5, S7 and S9 Tables. Co-expression analysis in Oncomine was used to identify sets of genes with synchronous expression patterns. The co-expression profiles of ACSL isoforms in different types of cancers were identified and presented as the pattern of heat map.

### Prognoscan database analysis

PrognoScan includes public microarray datasets with clinical annotation of gene expression and prognosis from Gene Expression Omnibus (GEO), ArrayExpress and individual laboratory web sites. The correlation between ACSL expression and survival in various types of cancers was analyzed by PrognoScan database (http://www.abren.net/PrognoScan/) [24]. The threshold was adjusted to cox p-value<0.05 and the analyses were summarized in S2, S4, S6, S8 and S10 Tables.

### Kaplan-Meier plotter database analysis

A Kaplan-Meier plotter database contains the 4,142 breast, 1,648 ovarian, 2,437 lung and 1,065 gastric cancer patients using probe sets on the HGU133 Plus 2.0 array. The correlation between ACSL expression and survival in breast, ovarian and lung was analyzed by Kaplan-Meier plotter (http://kmplot.com/analysis/) [25]. The hazard ratio with 95% confidence intervals and log rank p-value was also computed.

### GeneGo Metacore analysis

The function analysis was performed by GeneGo Metacore software using the GO Processes. We applied the co-expression profiles from the Oncomine as the parameter of correlation>0.6 and the top ten GO Processes were identified.

### Cell line

The HCT116 was obtained from Dr. H.L. Wu at National Cheng Kung University. The MDA-MB-231 was obtained from Dr. P.L Kuo at Kaohsiung Medical University. The A549 was obtained from Dr. W.T. Chang at National Cheng Kung University.

### RNA interference and lentivirus production

ACSL1 shRNAs were obtained from the National RNAi Core facility (Academia Sinica, Taipei, Taiwan). The following target sequences were used: GCCCAGATGATACTTTGATAT and CCCTTGGTGTATTTCTATGAT. The Turbofect transfection reagent was used for production of lentiviral particles according to the protocol provided from the National RNAi Core facility.

### Western blotting analysis

Cell lysate was harvested in RIPA lysis buffer and the amount of protein was determined with Micro BCA Protein Assay kit (Millipore, MA, USA). 30μg protein lysate was loaded into acrylamide gels and then transferred onto polyvinylidene fluoride membranes (Amersham Biosciences, Piscataway, NJ). The membrane was blocked with 5% nonfat dry milk and incubated with primary antibody for specific protein overnight. The membranes were incubated with horseradish peroxidase-conjugated secondary antibody and probed with ECL western blotting detection system (Millipore, MA, USA) and visualized with the BioSpectrum AC imaging system. The catalog number of antibody was as the following: the anti-ACSL1 antibody (#4047, Cell Signaling) and the anti-β-actin antibody (GTX109639, GeneTex).

### MTT assay

The cells were seeded onto 24 well plate at the density of 2X10^4^ per well. The cells were incubated with MTT (Thiazolyl Blue Tetrazolium Bromide, Sigma Chemical) for 3 hours at the 5% CO_2_ and 37°C and the absorbance was detected at 570 nm by ELISA reader.

### Boyden chamber assay

The upper wells of the chamber were seeded at the density of 2.6X10^4^ cells in serum-free DMEM, while lower wells contained the DMEM with 10% FBS. The cells of HCT116, A549 and MDA-MB-231 were incubated for 48, 24 and 6 hours, respectively. The cells that migrated through the polycarbonate filer were fixed using methanol and stained with Giemsa stain. The stained cell images were taken under the 10X objective lens by microscope and the mean number of stained cell was counted in five fields.

### Anchorage-independent growth

5X10^3^ cells were suspended in the 0.3% agar over a layer of 0.6% agar and incubated for 14 days in a 5% CO_2_ atmosphere humidified incubator at 37°C. The colonies were stained with 0.05% crystal violet. The colony images were taken under the 4X objective lens by microscope and the mean area of stained colony was quantified in ten fields by the Image-Pro Plus software.

### Statistical analysis

All statistical analyses were performed using GraphPad Prism version 4 (GraphPad Software, La Jolla, CA, USA). The statistical analysis was performed using two-way ANOVA followed by Bonferroni post-tests for MTT assay and one-way ANOVA followed by Tukey post hoc for migration and soft agar assay.

## Result

**Fig 1:**
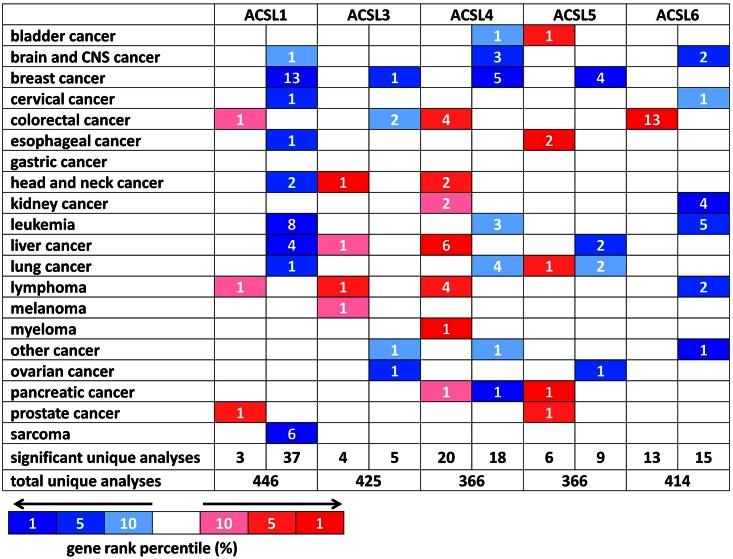
ACSL mRNA expression in various cancer types. The comparison indicated the number of datasets with ACSL mRNA overexpression (right column, red) and under expression (left column, blue) in cancer versus normal tissue. The threshold was designed with following parameters: p-value of 1E-4, fold change of 2, and gene ranking of 10%.

ACSL contributes to the different physiological roles in various types of cancers [12]. ACSL1 modulates the uptake of fatty acid in hepatoma cells [26]. Hepatocytic deletion of Pten in mice develops hepatocellular carcinoma and increased acsl5:acsl1 ratio [27]. To elucidate the role of ACSL family in cancer, the systematic analysis of ACSL family expression was demonstrated using Oncomine database. The expression of ACSL family was compared between tumor and normal tissues in different types of cancers. The threshold was designed as the following parameters: p-value of 1E-4, fold change of 2, and top gene ranking of 10%. The ACSL expression was higher in cancer than that in normal tissue in certain types of cancers. On the other hand, ACSL expression was lower in cancer than that in normal tissue in other types of cancers. These data indicates that individual ACSL may play either oncogenic or anti-oncogenic function depending on the cancer types (Fig 1). Therefore, detailed analysis of ACSL1, ACSL3, ACSL4, ACSL5, and ACSL6 were described below.

### ACSL1

**Fig 2:**
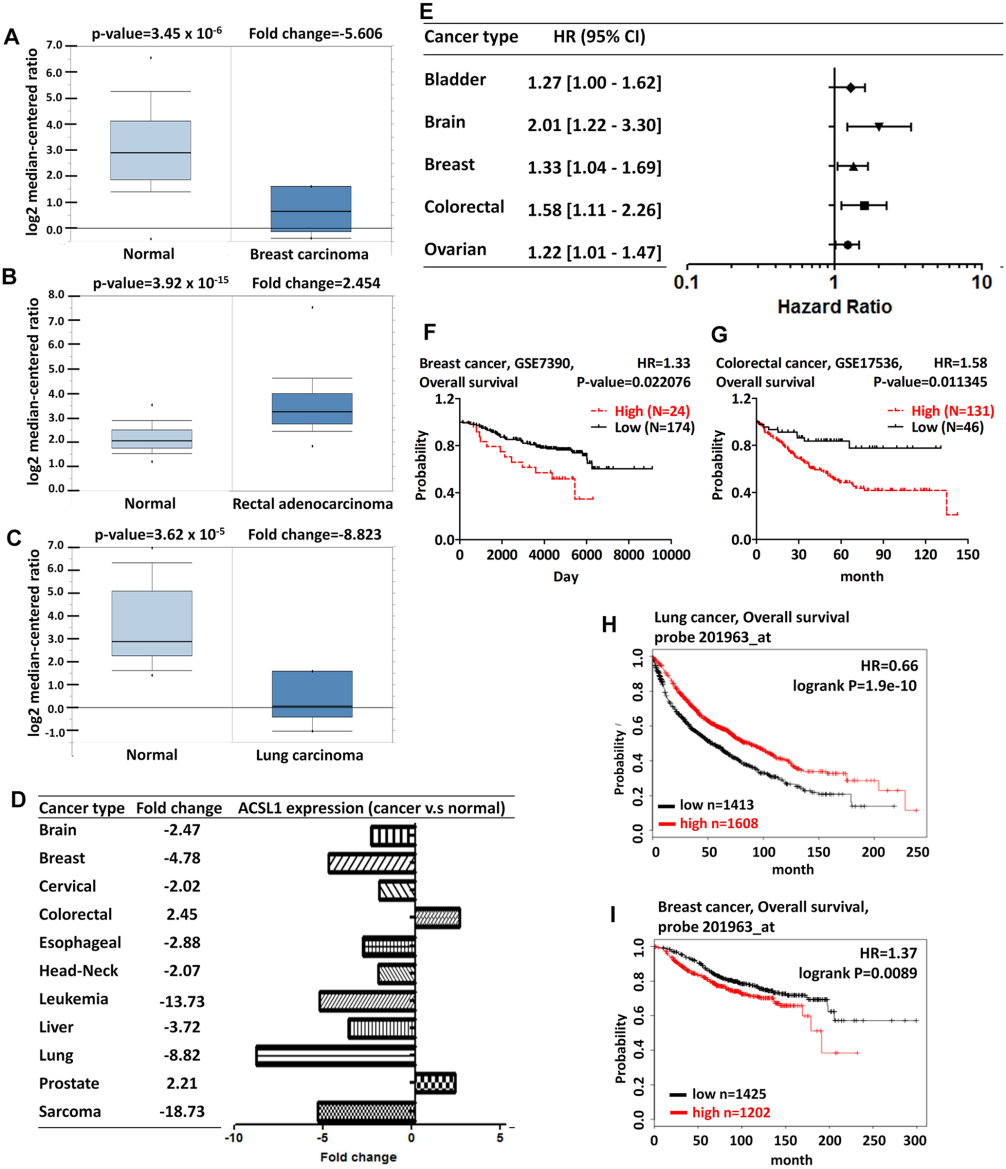
ACSL1 analysis in different cancer types. The box plot comparing specific ACSL1 expression in normal (left plot) and cancer tissue (right plot) was derived from Oncomine database. The analysis was shown in breast carcinoma relative to normal breast (A), in rectal adenocarcinoma relative to normal rectum (B), and in lung carcinoma relative to normal lung (C). The fold change of ACSL1 in various types of cancers was identified from our analyses in S1 Table and expressed as the forest plot (D). The statistically significant hazard ratio in various types of cancers was identified from our analyses in S2 Table and expressed as the forest plot (E). The survival curve comparing the patient with high (red) and low (black) expression in breast (F) and colorectal (G) cancer was identified as the threshold of cox p-value<0.05 from PrognoScan database. The survival curve comparing the patient with high (red) and low (black) expression in lung (H) and breast (I) cancer was plotted from Kaplan-Meier plotter database.

ACSL1 is associated with glucose homeostasis. The activation of ACSL1 regulates the insulin resistance by PKC activation in muscle cell [28,29]. ACSL1 interacts with the fatty acid transport protein (FATP), which is able to facilitate the uptake of fatty acid [30]. The activation of acyl-CoA-synthetase by ACSL1 promotes the fatty acid accumulation, indicating the potential target for hepatic steatosis [26,31]. Loss of ACSL1 favors the ABCA1 expression, contributing to the apoA-I-mediated cholesterol efflux in macrophage [11]. Lacking ACSL1 in heart-specific tissue drives the reduction of β-oxidation and results in the heart dysfunction [32]. In addition, miR-205 blocks the lipogenesis in liver cancer and anti-miR-205 promotes the increase of triglyceride by ACSL1. The negative-correlation of miR-205 and ACSL1 expression in hepatitis B virus X protein (HBx)-transgenic mice suggests that miR-205 leads to the dysregulation of lipid metabolism by ACSL1 and the cancer progression [33]. We applied Oncomine database to identify the ACSL1 expression in various kinds of cancer with the thresholds mentioned above. ACSL1 was upregulated in colorectal cancer, but decreased in lung and breast cancer (Fig 2A–2C). In addition, there was a lower expression level of ACSL1 in brain, cervical, esophageal, head and neck, leukemia, liver, and sarcoma cancers (Fig 2D and S1 Table). To further elucidate the role of ACSL1 in cancer progression, the prognostic value of ACSL1 expression in bladder, brain, breast, colorectal and ovarian cancer patients was determined using PrognoScan database according to the parameter of cox p-value<0.05 (Fig 2E and S2 Table). The breast and colorectal cancer patients with higher ACSL1 expression has poor survival (Fig 2F and 2G). We further used the Kaplan-Meier plotter database to evaluate the survival of breast and lung cancer patients. The probe 201963 for ACSL1 was used in analyzing the prognostic value in breast cancer and lung cancer patients. These data indicated that ACSL1 was associated with the poor survival in breast cancer, but was associated with the better survival in lung cancer (Fig 2H and 2I). The co-expression analysis reveals the biological function and provides the information for studying the underlying mechanism. We identified the co-expression profile of ACSL1 from the Oncomine database (S1A–S1C Fig). We further applied GeneGo Metacore to annotate gene ontology. No genes with the correlation > 0.6 were found in breast cancer dataset (S1A Fig). Specifically, the co-expression profiles for ACSL1 with a strong cluster of 126 genes (R > 0.6) across a panel of 65 rectal adenocarcinoma and 65 normal colorectal samples and 878 genes (R > 0.6) across a panel of 186 lung cancer and 17 normal lung samples were uploaded and the top ten GO Processes were listed (S1D and S1E Fig). In colorectal cancer, ACSL1 was coexpressed with myosin binding protein H (MYBPH), protein tyrosine phosphatase, receptor type E (PTPRE), 6-phosphofructo-2-kinase/fructose-2,6-biphosphatase 3 (PFKFB3), suppressor of cytokine signaling 3 (SOCS3), leukocyte immunoglobulin like receptors (LILRA3), and chemokine (C-C motif) ligand 4 (CCL4). These molecules mainly influence immune response, metabolism, and chemotaxis. As for lung cancer, ACSL1 was coexpressed with leucine rich repeat (in FLII) interacting protein 1 (LRRFIP1) and TSC22 domain family member 1 (TSC22D1). The analysis revealed that ACSL1 may be involved in immune response and cell chemotaxis in colorectal cancer and response to stress in lung cancer.

**Fig 3:**
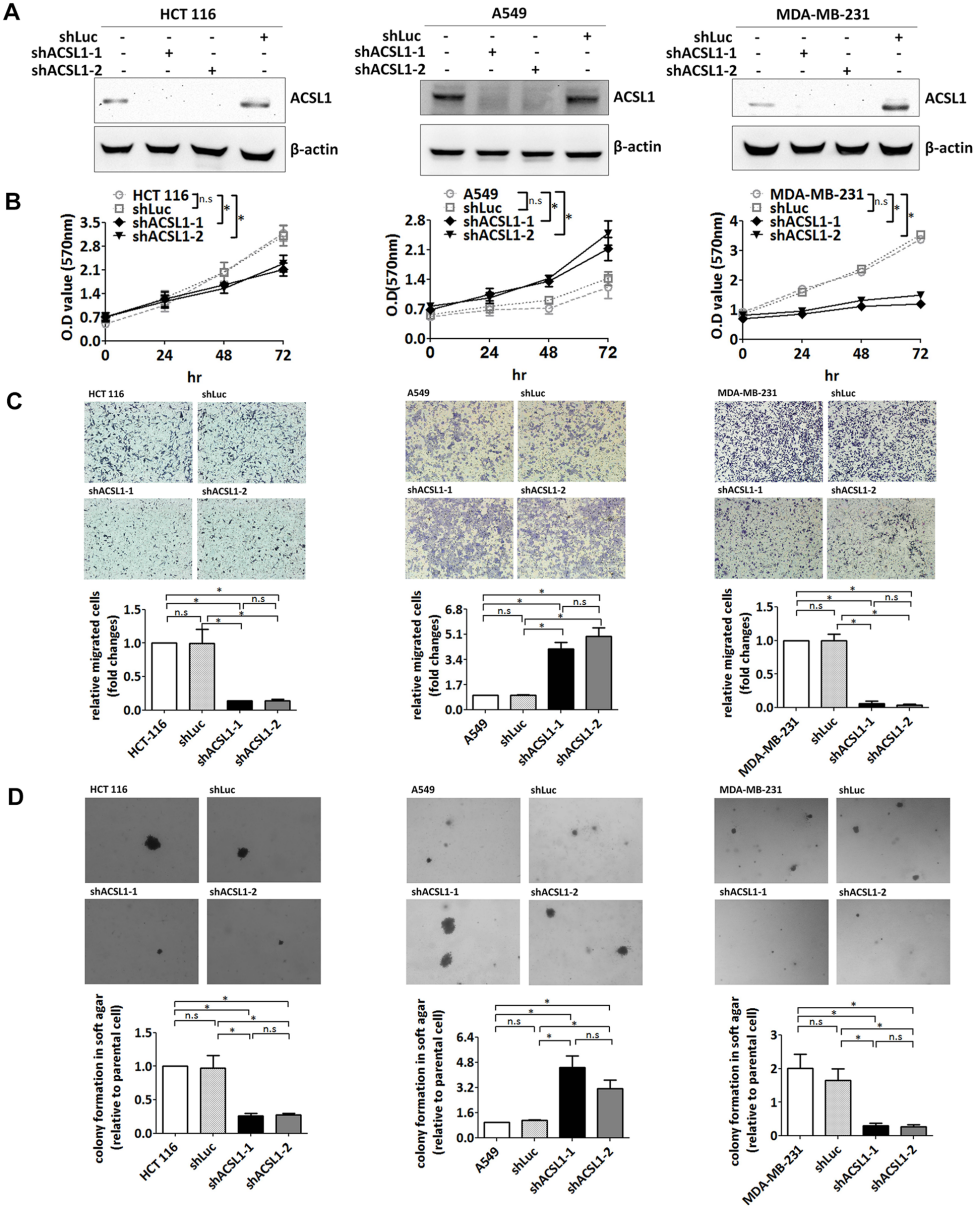
ACSL1 shRNAs modulate cancer growth *in vitro*. ACSL1 expression was downregulated with lentiviral particles containing shRNA targeting ACSL1 or luciferase in HCT116, A549 and MDA-MB-231. (A) The protein expression of ACSL1 was determined by western blotting with an anti-ACSL1 antibody. (B) The proliferation of the cell with ACSL1 knockdown expression was analyzed by MTT assay at the indicated time points. The statistical difference was calculated using two-way ANOVA followed by Bonferroni post-tests. * indicated p<0.05; n.s indicated not statistically significant. (C) The cell migration of the cell with ACSL1 knockdown expression was analyzed by Boyden chamber assay. The number of migrated cell was counted and the result was expressed as the fold change relative to the parental cell. All pairs of groups were compared using Tukey post hoc multicomparison test for one-way ANOVA. * indicated p<0.05; n.s indicated not statistically significant. (D) The cells with ACSL1 knockdown expression were plated in soft agar, and the colonies were monitored for 14 days. The colonies were quantified using Image-Pro Plus software. The result was expressed as the fold change relative to the parental cell. All pairs of groups were compared using Tukey post hoc multicomparison test for one-way ANOVA. * indicated p<0.05; n.s indicated not statistically significant.

Combination of the gene expression analysis from Oncomine and survival analysis from PrognoScan or Kaplan-Meier plotter revealed the oncogenic role of ACSL1 in colorectal cancer and the tumor suppressor role for ACSL1 in lung cancer. To validate the oncogenic role of ACSL1 in colorectal cancer and tumor suppressor role in lung cancer, two different lentiviral particles expressing ACSL1 shRNA were introduced to colorectal HCT116 cell line and lung A549 cell line, respectively. The knockdown efficacy of ACSL1 was determined by western blotting (Fig 3A). ACSL1 shRNA decreased proliferation in HCT116 cells, but increased proliferation in A549 cells (Fig 3B). In addition, the anchorage-independent growth and cell migration were inhibited in HCT116 after lentiviral particles expressing ACSL1 shRNA infection. In contrast, ACSL1 shRNA enhanced the anchorage-independent growth and cell migration in A549 (Fig 3C and 3D). The bioinformatics prediction of potential role of ACSL1 from Oncomine and PrognoScan in colorectal and lung cancer is supported by the *in vitro* assay.

Interestingly, the data obtained from Oncomine indicated that ACSL1 may function as a tumor suppressor gene in breast cancer (Fig 2A); however, the data obtained from PrognoScan and Kaplan-Meier plotter indicated that ACSL1 may play a potential oncogene in breast cancer (Fig 2F and 2I). To study the inconsistent bioinformatic results of ACSL1 in breast cancer, the lentiviral particles containing ACSL1 shRNAs were introduced to MDA-MB-231 breast cancer cell and the protein level of ACSL1 was determined by western blotting (Fig 3A, right panel). ACSL1 knockdown cell exhibited a reduced cell proliferation, which was demonstrated by MTT assay (Fig 3B, right panel). Downregulation of ACSL1 also inhibited anchorage-independent growth and cell migration of the MDA-MB-231 cells with soft agar assay and Boyden chamber assay (Fig 3C and 3D, right panel). Altogether, the prediction of oncogenic role of ACSL1 from PrognoScan and Kaplan-Meier plotter in breast cancer is supported by the *in vitro* experimental assay. The *in vitro* experimental analysis may be useful to resolve the discrepancy of analyses on ACSL1 obtained from Oncomine database and PrognoScan.

### ACSL3

**Fig 4:**
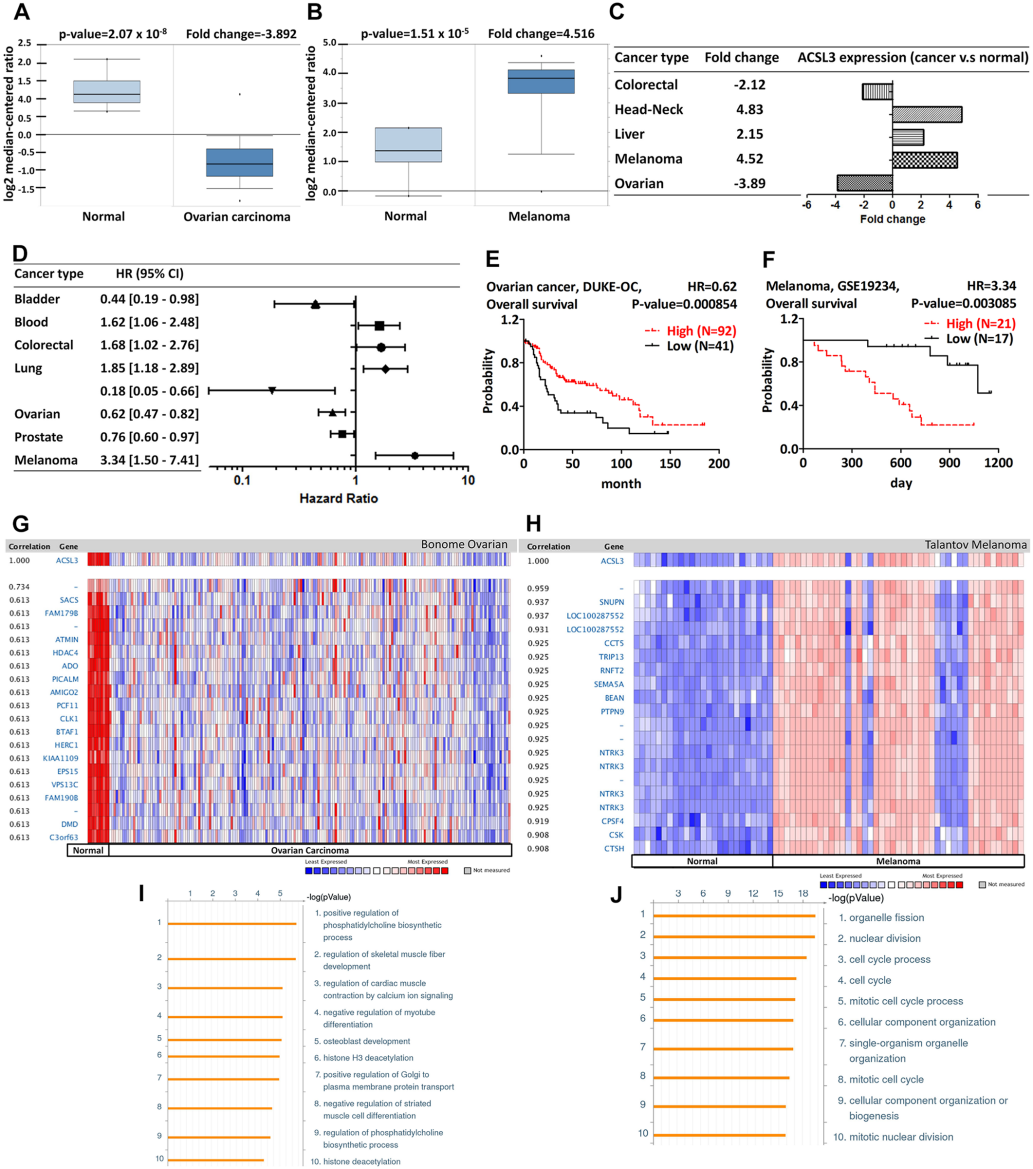
ACSL3 analysis in various cancer types. The box plot comparing specific ACSL3 expression in normal (left plot) and cancer tissue (right plot) was derived from Oncomine database. The analysis was shown in ovarian carcinoma relative to normal ovary (A) and in melanoma relative to normal skin (B). The fold change of ACSL3 in various types of cancers was identified from our analyses in S3 Table and expressed as the forest plot (C). The statistically significant hazard ratio in various types of cancers was identified from our analyses in S4 Table and expressed as the forest plot (D). The survival curve comparing the patient with high (red) and low (black) expression was plotted from PrognoScan database. The analysis of survival curve in ovarian cancer (E) and melanoma (F) was identified as the threshold of cox p-value<0.05. ACSL3 is coexpressed with the indicated genes across a panel of 185 ovarian carcinoma and 10 normal ovary tissues (G). ACSL3 is coexpressed with the indicated genes across a panel of 45 melanoma and 25 normal skin tissues (H). Top 10 significant GO processes were visualized by GeneGo Metacore software according to the co-expression profiles of the 27 genes in ovarian cancer (I) and 229 genes in melanoma (J). Bar length represented the significance and negative logarithm of enrichment p-value.

ACSL3 is abundant in lipid droplets and endoplasmic reticulum and is required for the fatty acid uptake. N-terminus of ACSL3 is required for the translocation from the endoplasmic reticulum to lipid droplet (LD) [34]. ACSL inhibitor, triacsin C, reduces ACSL3 expression and inhibits the lipid droplet formation [35,36]. ACSL3 is responsible for the VLDL assembly, which coordinate the lipid metabolism by exporting the exogenous cholesterol and triglyceride into plasma and is associated with the HCV infection. The downregulation of ACSL3 by siRNA reduces the VLDL secretion and inhibits the secretion of HCV particles from infected hepatocyte [37]. ACSL3 promotes palmitic acid-triggered osteoblastic gene expression and calcium deposition in vascular smooth muscle cell [38]. The cytokine oncostatin M-induced ACSL3 activation is dependent upon ERK-pathway activation and is associated with the decrease of cellular triglyceride and the increase of fatty acid β-oxidation [14]. Furthermore, PPAR-δ is involved in the activation of cytokine oncostatin M-induced ACSL3 expression in hepatoma [39]. The induction of ACSL3 is demonstrated by LXR, which is a nuclear protein and involved in the lipid uptake from lipoprotein [40]. Previous study indicates that ACSL3 is overexpressed in lung cancer [17]. In contrast, there is a lower ACSL3 expression in prostate cancer tissue compared to that in normal tissue [18]. Our analysis revealed that ACSL3 was down-regulated in ovarian cancer and up-regulated in melanoma (Fig 4A and 4B). In systematic analysis, ACSL3 was overexpressed in head-neck and liver cancer, but was underexpressed in colorectal cancer (Fig 4C and S3 Table). The prognostic value of ACSL3 was analyzed using the PrognoScan database with the threshold above (Fig 4D and S4 Table). The better prognosis in ovarian cancer patient and the worse prognosis in melanoma patient with higher ACSL3 expression were in accordance with the result from Oncomine (Fig 4E and 4F). The oncogenic role of ACSL3 in melanoma is consistent with a previous study [16]. On the other hand, ACSL3 may be considered as a potential tumor suppressor gene in ovarian cancer development. The co-expression profiles of ACSL3 were identified from Oncomine (Fig 4G and 4H). We identified the co-expression profiles for ACSL3 with a strong cluster of 27 genes across a panel of 185 ovarian carcinoma and 10 normal ovary tissues and 229 genes across a panel of 45 melanoma and 25 normal samples. GeneGo Metacore annotation for enriched biological process indicated that the genes involved in phosphatidylcholine biosynthetic process and organelle fission were more likely to be coexpressed in ovarian cancer and in melanoma, respectively (Fig 4I and 4J). It was interesting to note that ACSL3 was coexpressed with snurportin 1 (SNUPN), thyroid hormone receptor interactor 13 (TRIP13), and semaphorin 5A (SEMA5A). TRIP13 is involved in the spindle-assembly checkpoint and SNUPN is involved in snRNP importing. Semaphorin 5A (SEMA5A) is known to be responsible for certain types of autism.

### ACSL4

**Fig 5:**
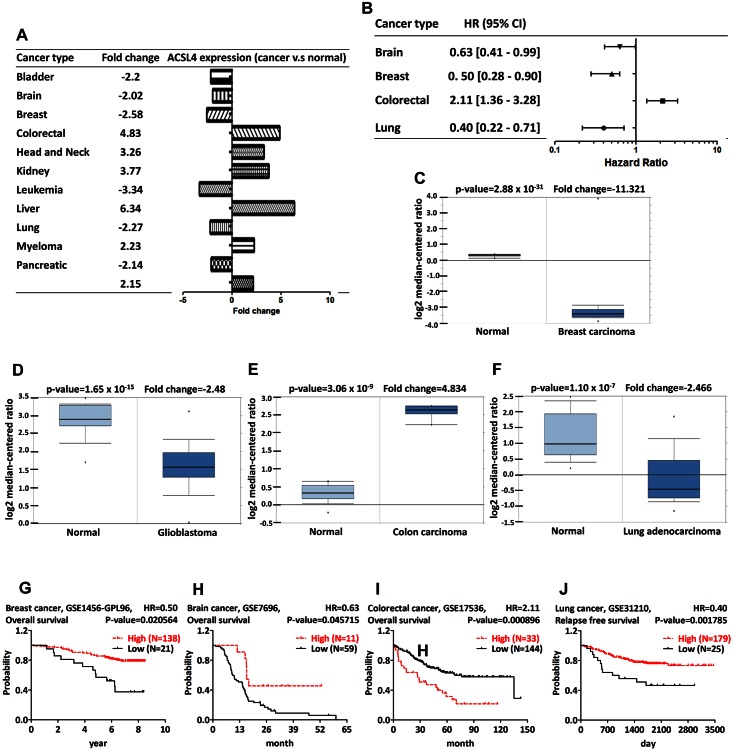
ACSL4 analysis in various cancer types. The fold change of ACSL4 in various types of cancers was identified from our analyses in S5 Table and expressed as the forest plot (A). The statistically significant hazard ratio in various types of cancers was identified from our analyses in S6 Table and expressed as the forest plot (B). The box plot comparing specific ACSL4 expression in normal (left plot) and cancer tissue (right plot) was derived from Oncomine database. The analysis was shown in breast carcinoma relative to normal breast (C), in glioblastoma relative to normal brain (D), in colorectal carcinoma relative to normal colorectal (E), and in lung adenocarcinoma relative to normal lung (F). The survival curve comparing the patient with high (red) and low (black) expression in breast cancer (G), brain cancer (H), colorectal cancer (I) and lung cancer (J) was plotted from PrognoScan database as the threshold of cox p-value<0.05.

ACSL4 is present in the peroxisome, mitochondria, and endoplasmic reticulum and is involved in arachidonate metabolism [41,42]. The deficiency of ACSL4 is correlated with the mental retardation and Alport syndrome [43]. The ACSL4-heterozygous mice have the abnormal uteri and uterine prostaglandin production [44]. ACSL4 regulates the neuron growth and differentiation [45]. Induction of ACSL4 expression promotes the tumor progression in xenograft model, and the combination of ACSL4, LOX-5 and COX-2 inhibitor effectively reduces the tumor formation *in vivo* [46]. The expression of ACSL4 mRNA is correlated with the estrogen receptor alpha expression and ACSL4 is sensitive to the triacsin C treatment, indicating that ACSL4 affects the steroid hormone-sensitivity in breast and prostate cancer [47]. ACSL4 catalyzes the conversion of free arachidonic acid into arachidonic acid-CoA ester and reduces the arachidonic acid-induced apoptosis in colon cancer. The overexpression of ACSL4 is associated with the colon carcinogenesis [48,49]. ACSL4 is also overexpressed in liver cancer tissues compared with the corresponding normal tissue. Arachidonic acid drives the ACSL4 ubiquitination by the substrate-induced posttranslational regulatory mechanism [50,51]. ACSL4 expression is negatively correlated with the amount of miR-205 in clinical HCC specimens. Hepatitis B virus X protein-induced lipogenesis can be abolished by miR-205 targeted ACSL4 mRNA [52]. Analysis from the Oncomine indicated that ACSL4 was down-regulated in bladder, brain, breast, leukemia, and lung cancer, but up-regulated in colorectal cancer, head and neck, kidney, myeloma, and liver cancer (Fig 5A and S5 Table). The prognostic analysis indicated that the colorectal patient with higher ACSL4 expression had poor survival; in contrast, the brain, breast, and lung cancer patient with lower ACSL4 expression had poor survival (Fig 5B and S6 Table). The analysis of ACSL4 gene expression in cancer from Oncomine (Fig 5C–5F) was in accordance with the survival analysis from PrognoScan (Fig 5G–5J). Combination of the gene expression from Oncomine and survival from PrognoScan revealed the oncogenic role of ACSL4 in colorectal cancer and the tumor suppressor role of ACSL4 in breast, brain, and lung cancer. These data are consistent with a previous study on the expression of ACSL4 in colon cancer tissues [49]. However, the results of the current analysis are inconsistent with a previous study investigating the overexpressed ACSL4 in breast cancer tissue [53]. The co-expression profiles for ACSL4 in breast, brain, colorectal and lung cancer were analyzed from Oncomine (S2A–S2D Fig). The co-expression profiles for ACSL4 were applied to annotate gene ontology using GeneGo Metacore with a strong cluster of 3,444 genes across a panel of 53 breast carcinoma and 6 normal breast samples, 117 genes across a panel of 10 brain tumor and 5 normal brain samples, 509 genes across a panel of 20 colorectal cancer and 20 normal colorectal samples, and 54 genes across a panel of 25 lung adenocarcinoma and 25 normal lung samples. The GO processes analyses demonstrated the co-expression genes, including serine protease inhibitor 2 (SERPIN2), heterogeneous nuclear ribonucleoprotein C-like 1 (HNRNPCL1), inter-alpha-trypsin inhibitor heavy chain 2 (ITIH2), protein C receptor (PROCR), and leucine rich repeat (in FLII) interacting protein 1 (LRRFIP1). These gene products were involved in cellular metabolic process, Fc receptor signaling pathway, extracellular matrix organization, and system development in breast, brain, colorectal and lung cancer (S2E–S2H Fig). It appears that ACSL4 may have distinct roles in various tissues. The detailed mechanism by which ACSL4 modulates cancer progression need to be further investigated.

### ACSL5

**Fig 6:**
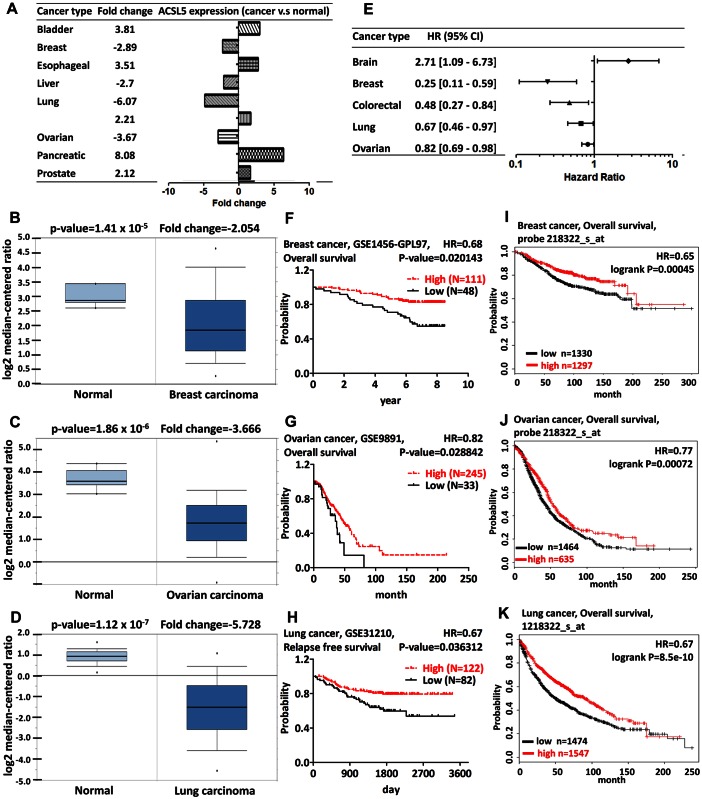
ACSL5 analysis in various cancer types. The fold change of ACSL5 in various types of cancers was identified from our analyses in S7 Table and expressed as the forest plot (A). The box plot comparing specific ACSL5 expression in normal (left plot) and cancer tissue (right plot) was derived from Oncomine database. The analysis was shown in breast carcinoma relative to normal breast (B), in ovarian carcinoma relative to normal ovary (C), and in lung carcinoma relative to normal lung (D). The statistically significant hazard ratio in various types of cancers was identified from our analyses in S8 Table and expressed as the forest plot (E). The survival curve comparing the patient with high (red) and low (black) expression in breast (F), ovarian (G), and lung (H) was plotted from PrognoScan database as the threshold of cox p-value<0.05. The survival curve comparing the patient with high (red) and low (black) expression in breast (I), ovarian (J) and lung (K) cancer was plotted from Kaplan-Meier plotter database.

ACSL5 is located on mitochondria and involved in the enterocyte apoptosis by alternative splicing [54]. A reduced ACSL5 expression is observed in the impaired small intestinal mucosa [55]. The expression of ACSL5 functions as a marker for gastrointestinal tract differentiation and villus atrophy [56,57]. ACSL5 is present in mitochondria and regulates lipid metabolism dependent on TP53 status in colorectal adenocarcinoma [58]. The lower expression of ACSL5 is observed in the colorectal cancer tissue and the patient with the lower ACSL5 has a longer disease-free interval (DFI) [59]. The expression level of ACSL5 is reduced in the neoplastic urothelial tissues and the expression pattern of ACSL5 is associated with the differential types of urothelium [60]. ACSL5 promotes the fatty acid uptake which results in fat storage or β-oxidation in liver cancer [14,61]. Up-regulation of ACSL5 is associated with the hepatocyte steatosis and is sensitive to fatty acid-induced hepatic cell death [62]. Downregulation of ACSL5 by RNAi significantly reduced fatty acid-induced lipid droplet formation in hepatocyte [63]. However, the impairment of ACSL activity in Acsl5-KO mice is insufficient to alter the long chain fatty acid absorption [64]. A single nucleotide polymorphism of ACSL5 promoter drives the ACSL5 mRNA expression in skeletal muscle cell and induces the weight loss [65]. ACSL5 is highly expressed in glioma and drives the cell growth through midkine (MDK) in the acidic microenvironment [66]. Our analysis revealed that ACSL5 is significantly overexpressed in bladder, esophageal, lung, pancreatic and prostate cancer. A significant decrease of ACSL5 was observed in breast, liver, lung, and ovarian cancer (Fig 6A–6D and S7 Table). There was a difference between lung carcinoma and lung adenocarcinoma that lung carcinoma expressed low level of ACSL5 and lung adenocarcinoma expressed high level of ACSL5 (S7 Table). To confirm the role of ACSL5 in cancer, we analyzed the prognostic value using PrognoScan. The analysis indicated that the brain cancer patient with higher ACSL5 expression had poor survival; in contrast, the patient with higher ACSL5 expression had good survival in breast, colorectal, lung and ovarian cancer (Fig 6E–6H and S8 Table). To further confirm the survival, the Kaplan-Meier plotter was used to identify the prognostic value in breast, lung and ovarian cancer. The better prognosis in breast, lung and ovarian cancer patient with higher ACSL5 expression was in accordance with the result from Oncomine and PrognoScan database, implying the tumor suppressor role of ACSL5 in breast, lung and ovarian cancer (Fig 6I–6K). The co-expression profiles of ACSL5 were identified from the Oncomine database (S3A–S3C Fig). The co-expression profiles for ACSL5 with a strong cluster of 3 genes across a panel of 40 breast carcinoma and 7 normal breast samples, 2 genes across a panel of 43 ovarian cancer and 10 normal ovary samples, and 111 genes across a panel of 91 lung cancer and 65 normal lung samples were uploaded to the Metacore and the top ten GO Processes were identified (S3D–S3F Fig). The top ranking genes included transmembrane protein 140 (TMEM140), TAP binding protein-like (TAPBPL), baculoviral IAP repeat containing 3 (BIRC3), protein tyrosine phosphatase, receptor type E (PTPRE), and serpin peptidase inhibitor clade B ovalbumin member 1 (SERPINB1). It is interesting to note that PTPRE is coexpressed with ACSL1 and ACSL5 in cancer, which warrants further investigation.

### ACSL6

**Fig 7:**
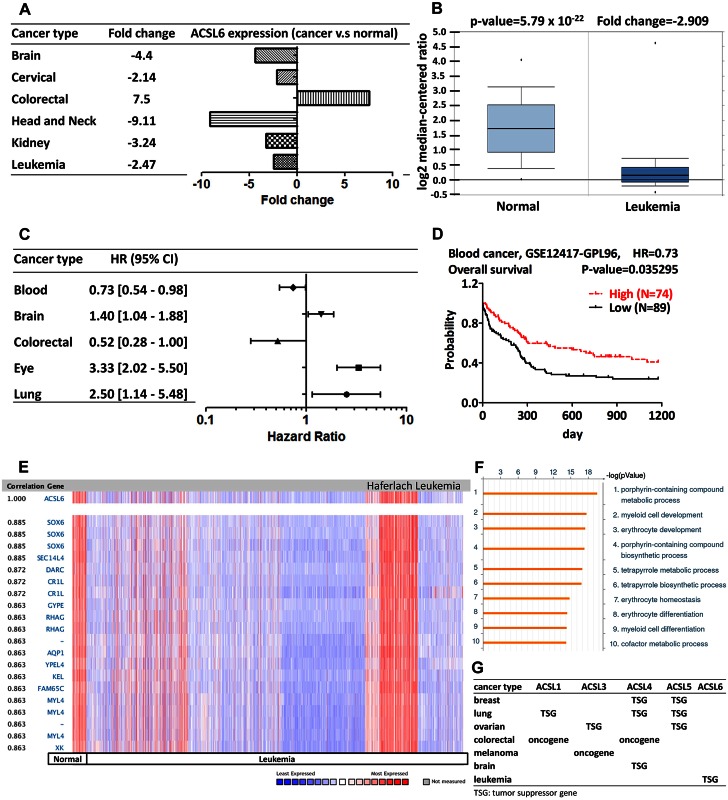
ACSL6 analysis in various cancer types. (A) The fold change of ACSL6 in various types of cancers was identified from our analyses in S9 Table and expressed as the forest plot. (B) The box plot comparing specific ACSL6 expression in normal (left plot) and cancer tissue (right plot) was derived from Oncomine database. The analysis was shown in acute myeloid leukemia relative to normal blood cell. (C) The statistically significant hazard ratio in various types of cancers was identified from our analyses in S10 Table and expressed as the forest plot. (D) The survival curve comparing the patient with high (red) and low (black) expression in blood cancer was plotted from PrognoScan database as the threshold of cox p-value<0.05. (E) ACSL6 is coexpressed with the indicated genes across a panel of 1,995 leukemia and 74 normal blood samples. (F) Top 10 significant GO processes were visualized by GeneGo Metacore software according to the co-expression profiles of the 144 genes in leukemia. Bar length represented the significance and negative logarithm of enrichment p-value. (G) The summary of predictive role of ACSL in different cancers is based on the consistent analyses of gene expression and outcome.

A linkage disequilibrium-based genome-wide association study reveals the role of ACSL6 in premature ovarian failure [67]. The schizophrenia is correlated with the single nucleotide polymorphism of ACSL6 [68]. The meta-analysis uncovers the association between ACSL6 and the number of cigarettes smoked per day. The nicotine-induced ACSL6 upregulation is restored by the nicotine receptor antagonist mecamylamine [69]. The murine long-chain acyl-CoA synthetase (mLACS) regulates the neuronal cell proliferation and differentiation [70]. Analysis from Oncomine revealed that the expression of ACSL6 was down-regulated in leukemia (Fig 7A). In addition, ACSL6 was decreased in most forms of cancers, except colorectal cancer (Fig 7B and S9 Table). The prognostic value of ACSL6 is analyzed using the PrognoScan database with the above threshold (Fig 7C and S10 Table). The better prognosis in leukemia patient was in accordance with the result from Oncomine database (Fig 7D). These data revealed that ACSL6 emerges as a potential tumor suppressor gene in leukemia. The co-expression profiles of ACSL6 were identified from Oncomine (Fig 7E). We identified the co-expression profiles for ACSL6 with a strong cluster of 144 genes across a panel of 1,995 leukemia and 74 normal blood tissues. ACSL6 gene was coexpressed with SRY (sex determining region Y)-box 6 (SOX6), Duffy blood group, chemokine receptor (DARC), and other genes involved in porphyrin-containing compound metabolic processes in leukemia cell (Fig 7F).

## Discussion

Increased fatty acid metabolism has been demonstrated to promote tumor progression in several types of cancers. Since ACSL family is required for complex lipid synthesis, the role of ACSL family is suggested to be pro-oncogenic previously [71]. However, the present report indicates that ACSL family members may function as tumor suppressor genes in a variety of cancers with bioinformatics analysis of cancer databases. Combination of the analyses from bioinformatics databases and *in vitro* analyses, ACSL is a tumor suppressor gene in ovarian and lung cancer and as an oncogene in colorectal cancer (Fig 7G). The analysis of genomic profile reveals that different types of cancers have their own genomic patterns and each cancer possess its biological characteristics. Previous studies demonstrate that ovarian and breast cancers display similar phenotypic and genotypic alterations. The alteration of PTEN and activation of PI3K pathway contribute to cell proliferation and metastasis in ovarian and breast cancer [72,73]. In addition, the breast and ovarian cancers display similar alteration of protein and lipid profiles by MALDI MS analysis [74]. The study assessing the genomic features of ovarian and breast cancer uncovers the similarity of molecular origin, and the dataset comparing the gene expression provides the evidence of similarity between basal-like breast cancer and squamous cell lung carcinoma [75,76]. Moreover, the analysis of gene expression predictors of response (GEPR) demonstrates the high similarity between ovary and lung carcinoma [77]. These evidences support our findings that ACSL may function as a tumor suppressor gene in ovarian and lung cancer. It should be noted that individual ACSL family members have substrate specificity and exert non-overlapping function. ACSL1 may be different from other ACSL family members, and play a pro-oncogenic role in breast cancer as demonstrated by PrognoScan and *in vitro* analysis. On the other hand, ACSL5 may exert its specific tumor suppressor role in different types of cancer through promoting ceramide [54] or Wnt2B palmitoylation [78,79].

The systematical analysis of lipidomic change shows that fatty acid (FA) and phosphatidylcholines (PC) are increased in various types of cancers and there is a higher ratio of monounsaturated fatty acid (MUFA)/saturated fatty acid (SFA) in breast, lung, colorectal, gastric, and esophageal cancer tissues compared to the adjacent normal tissues. Nevertheless, the ratio of PC(36:1)/PC(36:0) is increased in breast, lung, gastric, and esophageal cancer, but is not altered in colorectal cancer, indicating the cell type-specific metabolic features in colorectal cancer [80]. The network of ACSL1, ACSL4 and steraroyl-CoA desaturase-1 (SCD), which catalyzes the conversion of SFA into MUFA, is demonstrated to induce EMT program and the higher expression level of ACSL1/ACSL4/SCD is associated with the poorer survival outcome in colorectal cancer [81]. These studies describing the potential oncogenic role of ACSL and the differential genomic background in colorectal cancer are in concert with our analyses.

The genomic analysis reveals that different types of cancers have their own genomic patterns and each cancer possess its biological characteristics. Our data indicated that ACSL1 shRNA decreased cell growth and migration in HCT116, but increased cell growth and migration in A549 cells. The ACSL1-coexpressed genes were identified from colorectal and lung cancer datasets respectively. Among the strong cluster of 126 genes in colorectal cancer and 878 genes in lung cancer, only eight genes were co-expressed in both of clusters. In addition, the GO Process demonstrates the diverse effects of ACSL1 on colorectal and lung cancer. It is interesting to note that bioinformatics analysis suggests a potential tumor suppressor role of ACSL family in lung cancer. ACSL is required for conversion of fatty acid into complex lipid or β-oxidation. A report has indicated that PPAR-γ induces anti-proliferation through, at least in part, β-oxidation [82]. Overexpression of ACSL may increase the flux of β-oxidation pathway by PPAR-γ [83]. On the other hand, fatty acid β-oxidation may play an important oncogenic role in breast cancer [84,85]. These data implies that ACSL1 may behave oppositely when tumor growth has a different metabolic requirement.

In order to have the compelling analysis, we performed the analyses based on a large set of gene expression with clearly defined p-value, fold change and top 10% gene ranking and prognostic outcome with defined p-value and hazard ratio between cancer and normal tissues. With the aim of predicting the potential role of ACSL in cancers, we identified all of the results with statistical significance of gene expression from Oncomine in S1, S3, S5, S7 and S9 Tables and identified all of the results with statistical significance of survival outcome from PrognoScan in S2, S4, S6, S8 and S10 Tables. Most of the analyses predicted the same trend in one cancer. However, a few analyses gave the inconsistent trend in one cancer, including the outcome of ACSL3 in lung cancer of GSE13213 and GSE31210 datasets (Fig 4D and S4 Table), the expression of ACSL4 in Buchholz and Badea pancreatic cancer datasets (Fig 5A and S5 Table), and the expression of ACSL5 in Hou and Okayama lung cancer datasets (Fig 6A and S7 Table). These datasets were excluded from our analyses, and we defined the potential role of ACSL only based on the consistent analyses of gene expression and outcome. Our approach is less likely to generate the improper or designed data mining and provides the reasonable evaluation with a statistically unbiased assessment.

In summary, bioinformatic analysis with Oncomine and PrognoScan database indicates that ACSL family is involved in the cancer development. The design of inhibitor or activator of ACSL family for cancer therapy is dependent on cancer type since ACSL may play either oncogenic or tumor suppressor role in different types of cancer. In the future, the coexpressed genes with ACSL family identified in this study may be employed to investigate the signal network of ACSL family in cancer or other diseases.

## Supporting Information

S1 Fig Distribution of co-expression profiles and top ten GO processes for ACSL1. ACSL1 was coexpressed with the indicated genes across a panel of 532 breast cancer and 61 normal breast tissues (A), across a panel of 65 rectal adenocarcinoma and 65 normal colorectal tissues (B), and across a panel of 186 lung cancer and 17 normal lung tissues (C). Top 10 significant GO processes were visualized by GeneGo Metacore software according to the co-expression profiles of the 126 genes in colorectal cancer (D) and 878 genes in lung cancer (E). Bar length represented the significance and negative logarithm of enrichment p-value.
(TIF)

S2 Fig Distribution of co-expression profiles and top ten GO processes for ACSL4. ACSL4 was coexpressed with the indicated genes across a panel of 53 breast carcinoma and 6 normal breast tissues (A), across a panel of 10 brain cancer and 5 normal brain tissues (B), across a panel of 20 colorectal cancer and 20 normal colorectal tissues (C) and across a panel of 25 lung adenocarcinoma and 25 normal lung tissues (D). Top 10 significant GO processes were visualized by GeneGo Metacore software according to the co-expression profiles of the 3,444 genes in breast cancer (E), 117 genes in brain cancer (F), 509 genes in colorectal cancer (G), and 54 genes in lung cancer (H). Bar length represented the significance and negative logarithm of enrichment p-value.
(TIF)

S3 Fig Distribution of co-expression profiles and top ten GO processes for ACSL5. ACSL5 was coexpressed with the indicated genes across a panel of 40 breast carcinoma and 7 normal breast tissues (A), across a panel of 43 ovarian cancer and 10 normal ovary tissues (B), and across a panel of 91 lung cancer and 65 normal lung tissues (C). Top 10 significant GO processes were visualized by GeneGo Metacore software according to the co-expression profiles of the 3 genes in breast cancer (D), 2 genes in ovarian cancer (E), and 111 genes in lung cancer (F). Bar length represented the significance and negative logarithm of enrichment p-value.
(TIF)

S1 Table ACSL1 expression in cancers. (DOC)

S2 Table The association of ACSL1 expression and the survival in cancer patients. (DOC)

S3 Table ACSL3 expression in cancers. (DOC)

S4 Table The association of ACSL3 expression and the survival in cancer patients. (DOC)

S5 Table ACSL4 expression in cancers. (DOC)

S6 Table The association of ACSL4 expression and the survival in cancer patients. (DOC)

S7 Table ACSL5 expression in cancers. (DOC)

S8 Table The association of ACSL5 expression and the survival in cancer patients. (DOC)

S9 Table ACSL6 expression in cancers. (DOC)

S10 Table The association of ACSL6 expression and the survival in cancer patients. (DOC)

S11 Table The reference lists in supplementary tables. (DOC)
