# Supplementary figures and images for: Systematic Analysis of Gene Expression Alterations and Clinical Outcomes for Long-Chain Acyl-Coenzyme A Synthetase Family in Cancer
Source: PLoS One. 2016 May 12;11(5):e0155660. doi: 10.1371/journal.pone.0155660 (PMC4865206; doi:10.1371/journal.pone.0155660)

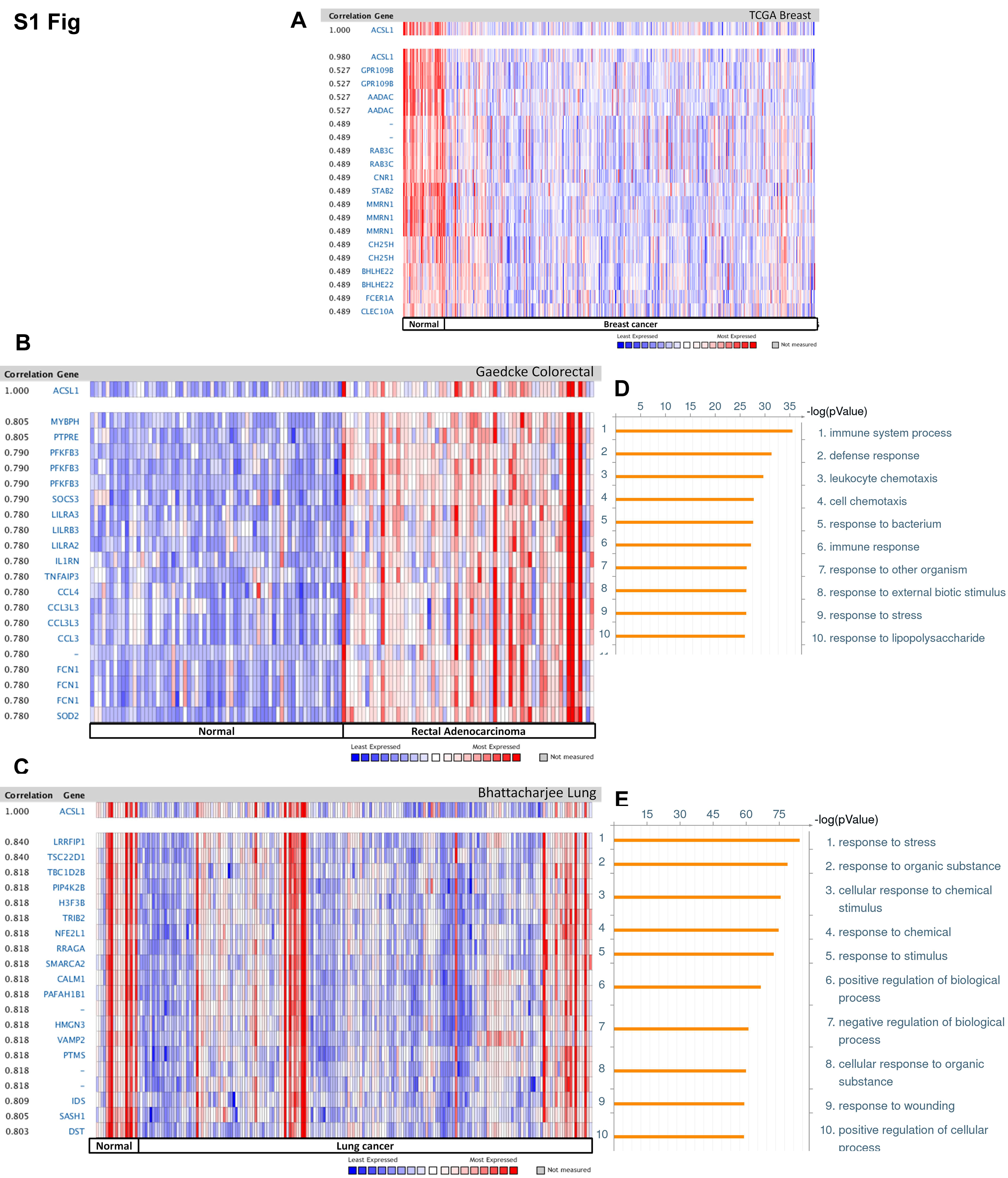

Supplement: S1 Fig — ACSL1 was coexpressed with the indicated genes across a panel of 532 breast cancer and 61 normal breast tissues (A), across a panel of 65 rectal adenocarcinoma and 65 normal colorectal tissues (B), and across a panel of 186 lung cancer and 17 normal lung tissues (C). Top 10 significant GO processes were visualized by GeneGo Metacore software according to the co-expression profiles of the 126 genes in colorectal cancer (D) and 878 genes in lung cancer (E). Bar length represented the significance and negative logarithm of enrichment p-value. (TIF) [file pone.0155660.s001.tif]

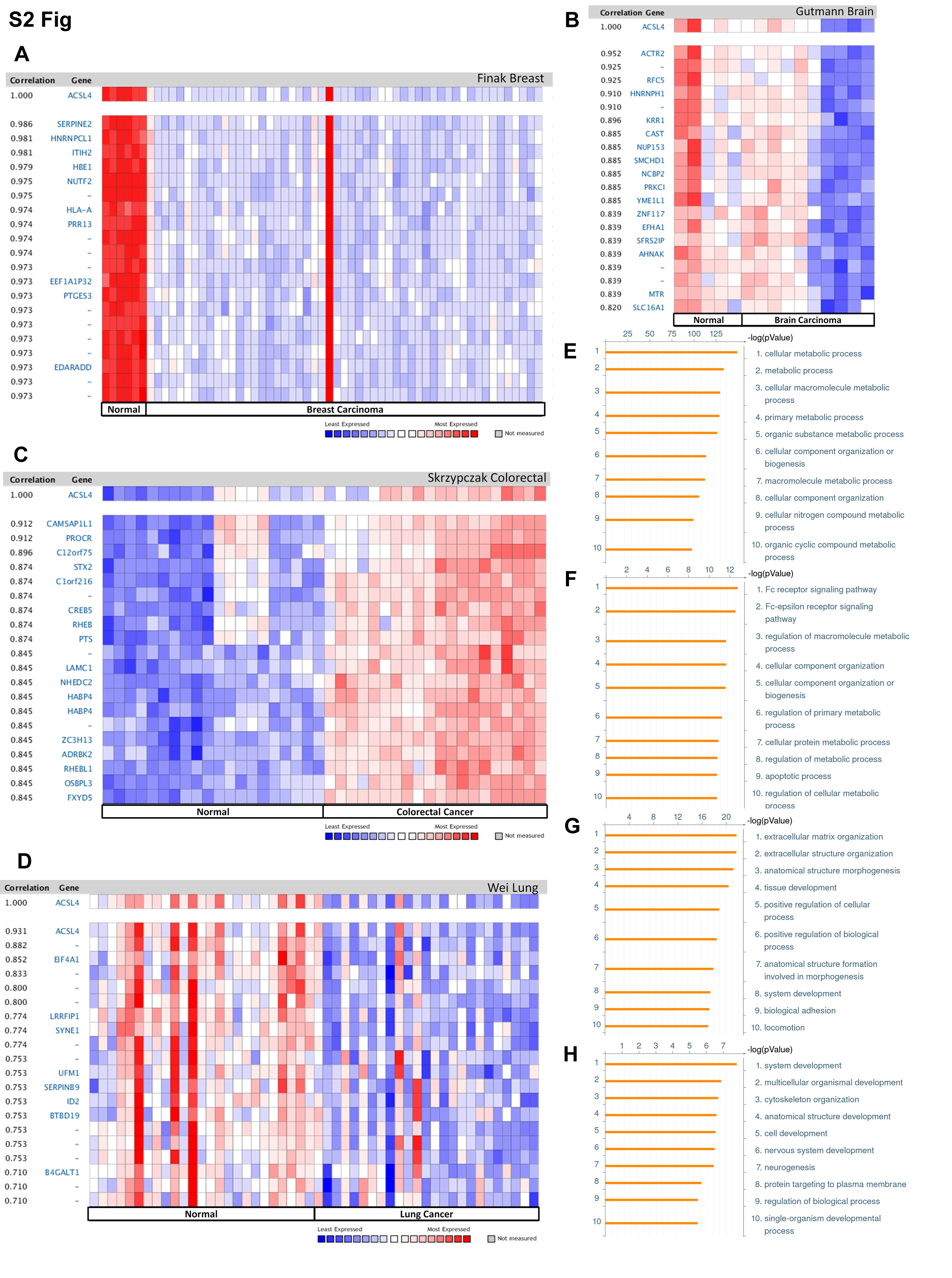

Supplement: S2 Fig — ACSL4 was coexpressed with the indicated genes across a panel of 53 breast carcinoma and 6 normal breast tissues (A), across a panel of 10 brain cancer and 5 normal brain tissues (B), across a panel of 20 colorectal cancer and 20 normal colorectal tissues (C) and across a panel of 25 lung adenocarcinoma and 25 normal lung tissues (D). Top 10 significant GO processes were visualized by GeneGo Metacore software according to the co-expression profiles of the 3,444 genes in breast cancer (E), 117 genes in brain cancer (F), 509 genes in colorectal cancer (G), and 54 genes in lung cancer (H). Bar length represented the significance and negative logarithm of enrichment p-value. (TIF) [file pone.0155660.s002.tif]

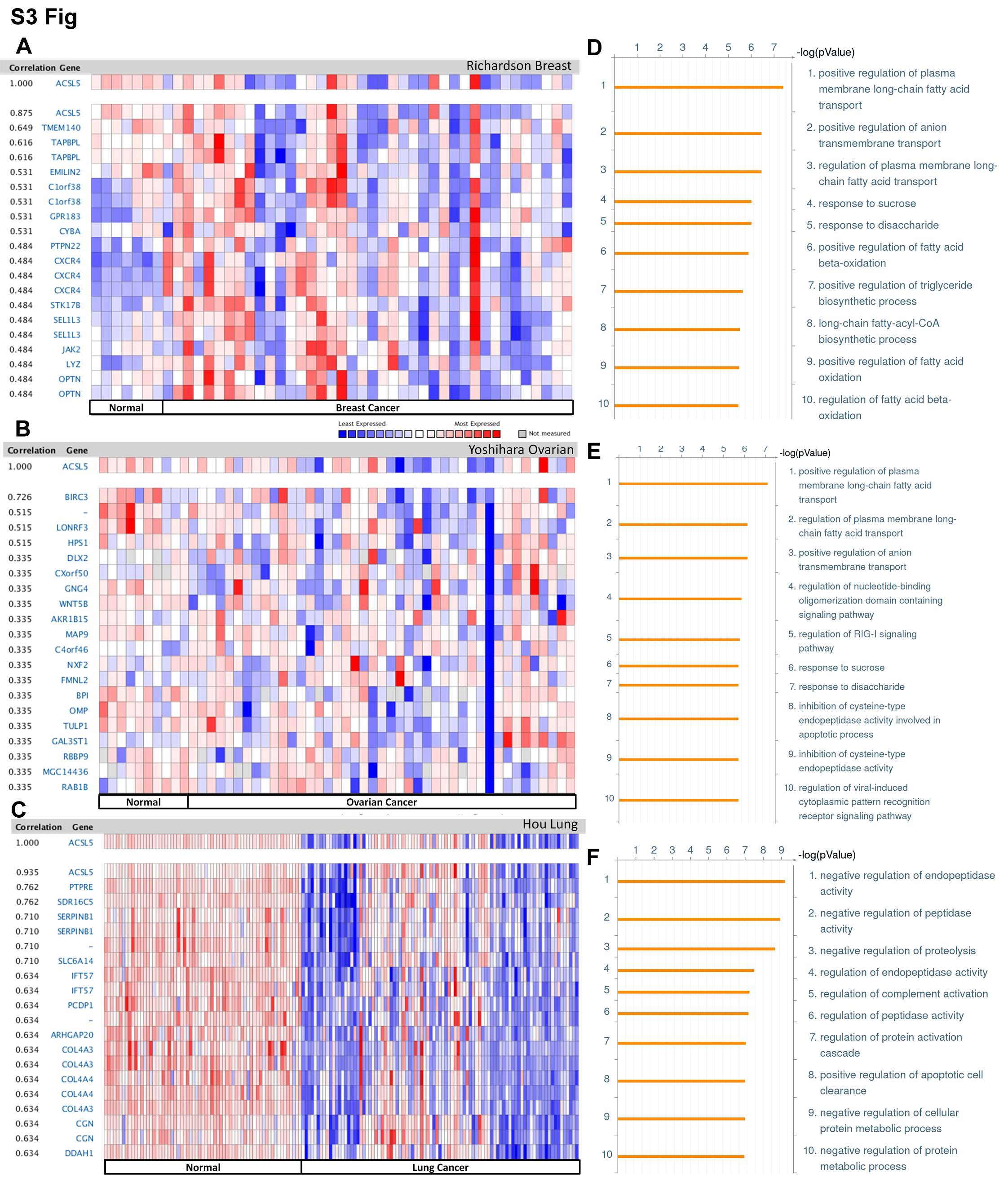

Supplement: S3 Fig — ACSL5 was coexpressed with the indicated genes across a panel of 40 breast carcinoma and 7 normal breast tissues (A), across a panel of 43 ovarian cancer and 10 normal ovary tissues (B), and across a panel of 91 lung cancer and 65 normal lung tissues (C). Top 10 significant GO processes were visualized by GeneGo Metacore software according to the co-expression profiles of the 3 genes in breast cancer (D), 2 genes in ovarian cancer (E), and 111 genes in lung cancer (F). Bar length represented the significance and negative logarithm of enrichment p-value. (TIF) [file pone.0155660.s003.tif]
