# Supplementary material for: Systematic Analysis of Gene Expression Alterations and Clinical Outcomes for Long-Chain Acyl-Coenzyme A Synthetase Family in Cancer
Source: PLoS One. 2016 May 12;11(5):e0155660. doi: 10.1371/journal.pone.0155660 (PMC4865206; doi:10.1371/journal.pone.0155660)
Supplement: S1 Table — (DOC) [file pone.0155660.s004.doc]

| **Supplementary Table 1. ACSL1 expression in cancers** | | | | | | |
| --- | --- | --- | --- | --- | --- | --- |
| **Cancer** | cancer subtype | p-value | fold change | rank (%) | sample | reference |
| **Brain** | Oligodendroglioma | 1.86E-09 | -2.47 | 9 | 180 |  |
|  |  |  |  |  |  |  |
| **Breast** | Ductal Breast Carcinoma | 2.94E-06 | -4.78 | 2 | 85 |  |
|  | Ductal Breast Carcinoma | 7.40E-05 | -4.52 | 3 | 167 |  |
|  | Invasive Lobular Breast Carcinoma | 7.78E-12 | -3.88 | 5 | 593 | TCGA |
|  | Invasive Breast Carcinoma | 1.19E-14 | -3.01 | 7 | 593 | TCGA |
|  | Breast Phyllodes Tumor | 4.78E-09 | -4.11 | 1 | 2136 |  |
|  | Medullary Breast Carcinoma | 1.53E-12 | -2.53 | 5 | 2136 |  |
|  | Breast Carcinoma | 1.65E-06 | -3.15 | 6 | 2136 |  |
|  | Mucinous Breast Carcinoma | 3.89E-15 | -2.43 | 7 | 2136 |  |
|  | Invasive Lobular Breast Carcinoma | 1.52E-22 | -2.74 | 8 | 2136 |  |
|  | Tubular Breast Carcinoma | 5.50E-17 | -2.56 | 9 | 2136 |  |
|  | Invasive Breast Carcinoma | 2.01E-05 | -2.07 | 10 | 2136 |  |
|  |  |  |  |  |  |  |
| **Cervical** | Cervical Squamous Cell Carcinoma | 2.29E-06 | -2.02 | 4 | 66 |  |
|  |  |  |  |  |  |  |
| **Colorectal** | Rectal Adenocarcinoma | 3.92E-15 | 2.45 | 10 | 130 |  |
|  |  |  |  |  |  |  |
| **Esophageal** | Esophageal Adenocarcinoma | 2.26E-15 | -2.88 | 5 | 118 |  |
|  |  |  |  |  |  |  |
| **Head-Neck** | Tongue Squamous Cell Carcinoma | 7.90E-06 | -2.07 | 5 | 58 |  |
|  | Salivary Gland Adenoid Cystic Carcinoma | 3.13E-05 | -6.55 | 5 | 22 |  |
|  |  |  |  |  |  |  |
| **Leukemia** | B-Cell Acute Lymphoblastic Leukemia | 7.13E-34 | -13.73 | 1 | 127 |  |
|  | Acute Myeloid Leukemia | 2.55E-11 | -5.28 | 1 | 127 |  |
|  | T-Cell Acute Lymphoblastic Leukemia | 2.72E-09 | -34.58 | 1 | 127 |  |
|  | B-Cell Acute Lymphoblastic Leukemia | 6.35E-37 | -4.16 | 4 | 2096 |  |
|  | T-Cell Acute Lymphoblastic Leukemia | 1.23E-36 | -4.07 | 4 | 2096 |  |
|  | B-Cell Childhood Acute Lymphoblastic Leukemia | 4.49E-28 | -3.00 | 6 | 2096 |  |
|  | Chronic Lymphocytic Leukemia | 1.20E-28 | -3.13 | 9 | 2096 |  |
|  | Pro-B Acute Lymphoblastic Leukemia | 1.15E-16 | -2.48 | 10 | 2096 |  |
|  |  |  |  |  |  |  |
| **Liver** | Hepatocellular Carcinoma | 2.06E-47 | -3.72 | 3 | 445 |  |
|  | Hepatocellular Carcinoma | 2.72E-24 | -3.21 | 1 | 197 |  |
|  | Hepatocellular Carcinoma | 1.26E-07 | -6.11 | 4 | 43 |  |
|  | Hepatocellular Carcinoma | 6.02E-09 | -3.28 | 1 | 75 |  |
|  |  |  |  |  |  |  |
| **Lung** | Small Cell Lung Carcinoma | 3.62E-05 | -8.82 | 5 | 203 |  |
|  |  |  |  |  |  |  |
| **Prostate** | Prostate Carcinoma | 3.45E-05 | 2.21 | 2 | 102 |  |
|  |  |  |  |  |  |  |
| **Sarcoma** | Leiomyosarcoma | 6.68E-11 | -18.73 | 1 | 158 |  |
|  | Dedifferentiated Liposarcoma | 3.64E-09 | -14.08 | 2 | 158 |  |
|  | Myxofibrosarcoma | 2.85E-09 | -16.57 | 2 | 158 |  |
|  | Pleomorphic Liposarcoma | 6.82E-07 | -5.40 | 3 | 158 |  |
|  | Myxoid/Round Cell Liposarcoma | 4.87E-08 | -6.53 | 3 | 158 |  |
