# Supplementary material for: Systematic Analysis of Gene Expression Alterations and Clinical Outcomes for Long-Chain Acyl-Coenzyme A Synthetase Family in Cancer
Source: PLoS One. 2016 May 12;11(5):e0155660. doi: 10.1371/journal.pone.0155660 (PMC4865206; doi:10.1371/journal.pone.0155660)
Supplement: S2 Table — (DOC) [file pone.0155660.s005.doc]

| **Supplementary Table 2. The association of ACSL1 expression and the survival in cancer patients** | | | | | | |
| --- | --- | --- | --- | --- | --- | --- |
| **Cancer type** | N | COX P-VALUE | HR | ENDPOINT | DATASET | PROBE ID |
| **Bladder** | 165 | 4.95E-02 | 1.27 | Overall Survival | GSE13507 | ILMN_1684585 |
| **Brain** | 50 | 1.65E-03 | 2.62 | Overall Survival | MGH-glioma | 40082_at |
|  | 74 | 5.94E-03 | 2.01 | Overall Survival | GSE4412-GPL96 | 201963_at |
|  | 74 | 3.36E-02 | 1.7 | Overall Survival | GSE4412-GPL96 | 207275_s_at |
| **Breast** | 198 | 2.21E-02 | 1.33 | Overall Survival | GSE7390 | 207275_s_at |
|  | 198 | 3.51E-02 | 1.35 | Overall Survival | GSE7390 | 201963_at |
| **Colorectal** | 177 | 1.90E-02 | 1.64 | Disease Specific Survival | GSE17536 | 201963_at |
|  | 177 | 4.67E-02 | 1.47 | Overall Survival | GSE17536 | 207275_s_at |
|  | 145 | 2.43E-03 | 2.23 | Disease Free Survival | GSE17536 | 207275_s_at |
|  | 177 | 1.13E-02 | 1.58 | Overall Survival | GSE17536 | 201963_at |
|  | 145 | 2.55E-03 | 2.25 | Disease Free Survival | GSE17536 | 201963_at |
|  | 177 | 4.66E-02 | 1.56 | Disease Specific Survival | GSE17536 | 207275_s_at |
| **Ovarian** | 185 | 6.44E-03 | 1.27 | Disease Free Survival | GSE26712 | 201963_at |
|  | 185 | 3.46E-02 | 1.22 | Overall Survival | GSE26712 | 201963_at |
|  | 185 | 2.65E-02 | 1.32 | Disease Free Survival | GSE26712 | 207275_s_at |
