# Supplementary material for: Systematic Analysis of Gene Expression Alterations and Clinical Outcomes for Long-Chain Acyl-Coenzyme A Synthetase Family in Cancer
Source: PLoS One. 2016 May 12;11(5):e0155660. doi: 10.1371/journal.pone.0155660 (PMC4865206; doi:10.1371/journal.pone.0155660)
Supplement: S3 Table — (DOC) [file pone.0155660.s006.doc]

| **Supplementary Table 3. ACSL3 expression in cancers** | | | | | | |
| --- | --- | --- | --- | --- | --- | --- |
| **Cancer** | cancer subtype | p-value | Fold  change | rank (%) | sample | Reference |
| **Colorectal** | Colon Adenoma | 3.36E-05 | -2.12 | 7 | 40 | [18] |
|  |  |  |  |  |  |  |
| **Head-Neck** | Salivary Gland Adenoid Cystic Carcinoma | 1.00E-09 | 4.83 | 1 | 22 | [9] |
|  |  |  |  |  |  |  |
| **Liver** | Hepatocellular Carcinoma | 1.40E-37 | 2.15 | 8 | 445 | [12] |
|  |  |  |  |  |  |  |
| **Melanoma** | Cutaneous Melanoma | 1.51E-05 | 4.52 | 7 | 70 | [19] |
|  |  |  |  |  |  |  |
| **Ovarian** | Ovarian Carcinoma | 2.07E-08 | -3.89 | 5 | 195 | [20] |
