# Supplementary material for: Systematic Analysis of Gene Expression Alterations and Clinical Outcomes for Long-Chain Acyl-Coenzyme A Synthetase Family in Cancer
Source: PLoS One. 2016 May 12;11(5):e0155660. doi: 10.1371/journal.pone.0155660 (PMC4865206; doi:10.1371/journal.pone.0155660)
Supplement: S4 Table — (DOC) [file pone.0155660.s007.doc]

| **Supplementary Table 4. The association of ACSL3 expression and the survival in cancer patients** | | | | | | |
| --- | --- | --- | --- | --- | --- | --- |
| **Cancer type** | N | COX P-VALUE | HR | ENDPOINT | DATASET | PROBE ID |
| **Bladder** | 30 | 4.57E-02 | 0.44 | Overall Survival | GSE5287 | 201662_s_at |
| **Blood** | 79 | 2.68E-02 | 1.62 | Overall Survival | GSE12417-GPL570 | 201662_s_at |
| **Colorectal** | 177 | 4.15E-02 | 1.68 | Overall Survival | GSE17536 | 201661_s_at |
|  | 55 | 2.32E-02 | 2.29 | Disease Free Survival | GSE17537 | 201661_s_at |
|  | 49 | 2.24E-02 | 3.04 | Disease Specific Survival | GSE17537 | 201661_s_at |
| **Lung** | 117 | 7.24E-03 | 1.85 | Overall Survival | GSE13213 | A_24_P37319 |
|  | 204 | 3.81E-02 | 2.86 | Overall Survival | GSE31210 | 201662_s_at |
|  | 204 | 9.63E-03 | 0.18 | Overall Survival | GSE31210 | 201661_s_at |
|  | 204 | 4.20E-05 | 0.14 | Relapse Free Survival | GSE31210 | 201661_s_at |
| **Ovarian** | 133 | 3.99E-02 | 0.78 | Overall Survival | DUKE-OC | 201661_s_at |
|  | 133 | 8.54E-04 | 0.62 | Overall Survival | DUKE-OC | 201662_s_at |
| **Prostate** | 281 | 2.53E-02 | 0.76 | Overall Survival | GSE16560 | DAP3_3920 |
| **Skin** | 38 | 5.35E-02 | 2.25 | Overall Survival | GSE19234 | 201661_s_at |
|  | 38 | 3.08E-03 | 3.34 | Overall Survival | GSE19234 | 201662_s_at |
