# Supplementary material for: Systematic Analysis of Gene Expression Alterations and Clinical Outcomes for Long-Chain Acyl-Coenzyme A Synthetase Family in Cancer
Source: PLoS One. 2016 May 12;11(5):e0155660. doi: 10.1371/journal.pone.0155660 (PMC4865206; doi:10.1371/journal.pone.0155660)
Supplement: S5 Table — (DOC) [file pone.0155660.s008.doc]

| **Supplementary Table 5. ACSL4 expression in cancers** | | | | | | |
| --- | --- | --- | --- | --- | --- | --- |
| **Cancer** | cancer subtype | p-value | fold change | rank (%) | sample | Reference |
| **Bladder** | Superficial Bladder Cancer | 1.28E-08 | -2.20 | 8 | 157 | [21] |
|  |  |  |  |  |  |  |
| **Brain** | Glioblastoma | 1.86E-11 | -2.02 | 2 | 54 | [22] |
|  | Oligodendroglioma | 5.14E-13 | -2.23 | 3 | 180 | [1] |
|  | Glioblastoma | 1.65E-15 | -2.48 | 7 | 180 | [1] |
|  |  |  |  |  |  |  |
| **Breast** | Invasive Breast Carcinoma | 2.88E-31 | -11.32 | 1 | 59 | [23] |
|  | Invasive Lobular Breast Carcinoma | 4.51E-47 | -2.20 | 2 | 2136 | [4] |
|  | Mucinous Breast Carcinoma | 7.80E-19 | -2.58 | 5 | 2136 | [4] |
|  | Invasive Ductal Breast Carcinoma | 1.68E-49 | -2.12 | 5 | 2136 | [4] |
|  | Invasive Ductal Breast Carcinoma | 3.43E-25 | -2.42 | 6 | 593 | TCGA |
|  |  |  |  |  |  |  |
| **Colorectal** | Colon Carcinoma | 3.06E-09 | 4.83 | 2 | 40 | [18] |
|  | Colorectal Carcinoma | 1.84E-10 | 2.42 | 2 | 105 | [18] |
|  | Rectal Adenocarcinoma | 3.53E-25 | 2.29 | 3 | 130 | [6] |
|  | Colon Mucinous Adenocarcinoma | 8.58E-10 | 2.03 | 5 | 237 | TCGA |
|  |  |  |  |  |  |  |
| **Head and Neck** | Head and Neck Squamous Cell Carcinoma | 9.93E-10 | 3.26 | 3 | 54 | [24] |
|  | Oral Cavity Squamous Cell Carcinoma | 5.13E-10 | 2.08 | 5 | 79 | [25] |
|  |  |  |  |  |  |  |
| **Kidney** | Papillary Renal Cell Carcinoma | 7.18E-07 | 3.77 | 9 | 92 | [26] |
|  | Renal Pelvis Urothelial Carcinoma | 3.11E-05 | 3.60 | 10 | 92 | [26] |
|  |  |  |  |  |  |  |
| **Leukemia** | Acute Myeloid Leukemia | 1.35E-08 | -3.34 | 3 | 127 | [10] |
|  | B-Cell Acute Lymphoblastic Leukemia | 1.85E-08 | -3.60 | 7 | 127 | [10] |
|  | T-Cell Acute Lymphoblastic Leukemia | 2.85E-06 | -4.10 | 8 | 127 | [10] |
|  |  |  |  |  |  |  |
| **Liver** | Hepatocellular Carcinoma | 3.84E-73 | 9.87 | 1 | 445 |  |
|  | Hepatocellular Carcinoma | 1.77E-07 | 6.97 | 4 | 43 |  |
|  | Hepatocellular Carcinoma | 7.72E-07 | 3.95 | 7 | 115 | [27] |
|  | Hepatocellular Carcinoma | 4.39E-10 | 6.34 | 1 | 75 |  |
|  |  |  |  |  |  |  |
| **Lung** | Lung Adenocarcinoma | 9.05E-17 | -2.27 | 2 | 156 | [28] |
|  | Large Cell Lung Carcinoma | 4.35E-14 | -3.06 | 2 | 156 | [28] |
|  | Squamous Cell Lung Carcinoma | 4.73E-11 | -2.69 | 9 | 156 | [28] |
|  | Lung Adenocarcinoma | 3.85E-16 | -2.27 | 5 | 116 | [29] |
|  | Lung Adenocarcinoma | 1.10E-7 | -2.47 | 8 | 50 | [30] |
|  |  |  |  |  |  |  |
| **Myeloma** | Smoldering Myeloma | 3.63E-12 | 2.23 | 1 | 78 | [31] |
|  |  |  |  |  |  |  |
| **Pancreatic** | Pancreatic Ductal Adenocarcinoma | 1.25E-05 | -2.14 | 1 | 38 | [32] |
|  | Pancreatic Ductal Adenocarcinoma | 1.63E-08 | 2.15 | 8 | 78 | [33] |
