# Supplementary material for: Systematic Analysis of Gene Expression Alterations and Clinical Outcomes for Long-Chain Acyl-Coenzyme A Synthetase Family in Cancer
Source: PLoS One. 2016 May 12;11(5):e0155660. doi: 10.1371/journal.pone.0155660 (PMC4865206; doi:10.1371/journal.pone.0155660)
Supplement: S6 Table — (DOC) [file pone.0155660.s009.doc]

| **Supplementary Table 6. The association of ACSL4 expression and the survival in cancer patients** | | | | | | |
| --- | --- | --- | --- | --- | --- | --- |
| **Cancer type** | N | COX P-VALUE | HR | ENDPOINT | DATASET | PROBE ID |
| **Brain** | 70 | 4.57E-02 | 0.63 | Overall Survival | GSE7696 | 202422_s_at |
| **Breast** | 286 | 4.32E-02 | 1.44 | Distant Metastasis Free Survival | GSE2034 | 202422_s_at |
|  | 159 | 1.59E-02 | 0.43 | Disease Specific Survival | GSE1456-GPL96 | 202422_s_at |
|  | 159 | 2.06E-02 | 0.5 | Overall Survival | GSE1456-GPL96 | 202422_s_at |
| **Colorectal** | 177 | 8.78E-03 | 1.96 | Disease Specific Survival | GSE17536 | 202422_s_at |
|  | 177 | 8.96E-04 | 2.11 | Overall Survival | GSE17536 | 202422_s_at |
| **Lung** | 204 | 1.78E-03 | 0.4 | Relapse Free Survival | GSE31210 | 202422_s_at |
