# Supplementary material for: Systematic Analysis of Gene Expression Alterations and Clinical Outcomes for Long-Chain Acyl-Coenzyme A Synthetase Family in Cancer
Source: PLoS One. 2016 May 12;11(5):e0155660. doi: 10.1371/journal.pone.0155660 (PMC4865206; doi:10.1371/journal.pone.0155660)
Supplement: S7 Table — (DOC) [file pone.0155660.s010.doc]

| **Supplementary Table 7. ACSL5 expression in cancers** | | | | | | |
| --- | --- | --- | --- | --- | --- | --- |
| **Cancer** | cancer subtype | p-value | fold change | rank (%) | sample | Reference |
| **Bladder** | Superficial Bladder Cancer | 1.20E-14 | 3.81 | 5 | 157 | [21] |
|  |  |  |  |  |  |  |
| **Breast** | Invasive Ductal Breast Carcinoma | 2.13E-53 | -2.89 | 1 | 593 | TCGA |
|  | Invasive Breast Carcinoma | 3.99E-13 | -2.16 | 8 | 593 | TCGA |
|  | Mucinous Breast Carcinoma | 3.29E-15 | -2.10 | 7 | 2136 | [4] |
|  | Ductal Breast Carcinoma | 1.41E-05 | -2.05 | 7 | 47 | [34] |
|  |  |  |  |  |  |  |
| **Esophageal** | Esophageal Adenocarcinoma | 6.18E-14 | 3.51 | 3 | 118 | [7] |
|  |  |  |  |  |  |  |
| **Liver** | Hepatocellular Carcinoma | 2.96E-33 | -2.70 | 5 | 445 | [12] |
|  | Hepatocellular Carcinoma | 4.82E-06 | -3.81 | 6 | 43 | [12] |
|  |  |  |  |  |  |  |
| **Lung** | Squamous Cell Lung Carcinoma | 5.59E-13 | -6.07 | 7 | 156 | [28] |
|  | Large Cell Lung Carcinoma | 1.12E-07 | -5.73 | 9 | 156 | [28] |
|  | Lung Adenocarcinoma | 2.82E-14 | 2.21 | 3 | 246 | [35] |
|  |  |  |  |  |  |  |
| **Ovarian** | Ovarian Serous Cystadenocarcinoma | 1.86E-06 | -3.67 | 3 | 594 | TCGA |
|  |  |  |  |  |  |  |
| **Pancreatic** | Pancreatic Adenocarcinoma | 7.63E-06 | 8.08 | 1 | 36 | [36] |
| **Prostate** | Prostate Carcinoma | 8.24E-09 | 2.12 | 2 | 122 | [37] |
