# Supplementary material for: Systematic Analysis of Gene Expression Alterations and Clinical Outcomes for Long-Chain Acyl-Coenzyme A Synthetase Family in Cancer
Source: PLoS One. 2016 May 12;11(5):e0155660. doi: 10.1371/journal.pone.0155660 (PMC4865206; doi:10.1371/journal.pone.0155660)
Supplement: S8 Table — (DOC) [file pone.0155660.s011.doc]

| **Supplementary Table 8. The association of ACSL5 expression and the survival in cancer patients** | | | | | | |
| --- | --- | --- | --- | --- | --- | --- |
| **Cancer type** | N | COX P-VALUE | HR | ENDPOINT | DATASET | PROBE ID |
| **Brain** | 74 | 3.13E-02 | 2.71 | Overall Survival | GSE4412-GPL96 | 218322_s_at |
| **Breast** | 115 | 3.04E-02 | 0.36 | Distant Metastasis Free Survival | GSE19615 | 222592_s_at |
|  | 115 | 3.07E-02 | 0.25 | Distant Metastasis Free Survival | GSE19615 | 218322_s_at |
|  | 87 | 1.21E-02 | 0.61 | Distant Metastasis Free Survival | GSE6532-GPL570 | 218322_s_at |
|  | 87 | 1.21E-02 | 0.61 | Relapse Free Survival | GSE6532-GPL570 | 218322_s_at |
|  | 286 | 1.25E-02 | 0.57 | Distant Metastasis Free Survival | GSE2034 | 218322_s_at |
|  | 159 | 1.13E-03 | 0.19 | Disease Specific Survival | GSE1456-GPL96 | 218322_s_at |
|  | 159 | 1.49E-03 | 0.25 | Overall Survival | GSE1456-GPL96 | 218322_s_at |
|  | 159 | 1.68E-03 | 0.25 | Relapse Free Survival | GSE1456-GPL96 | 218322_s_at |
|  | 159 | 2.01E-02 | 0.68 | Overall Survival | GSE1456-GPL97 | 222592_s_at |
|  | 159 | 4.84E-02 | 0.68 | Disease Specific Survival | GSE1456-GPL97 | 222592_s_at |
|  | 54 | 5.24E-03 | 0.06 | Disease Free Survival | GSE7378 | 218322_s_at |
|  | 236 | 4.49E-02 | 0.54 | Disease Specific Survival | GSE3494-GPL96 | 218322_s_at |
| **Colorectal** | 62 | 1.10E-02 | 0.48 | Overall Survival | GSE12945 | 218322_s_at |
|  | 177 | 4.06E-02 | 0.67 | Disease Specific Survival | GSE17536 | 222592_s_at |
|  | 145 | 1.25E-02 | 0.53 | Disease Free Survival | GSE17536 | 222592_s_at |
|  | 226 | 3.17E-03 | 0.57 | Disease Free Survival | GSE14333 | 222592_s_at |
| **Lung** | 204 | 3.63E-02 | 0.67 | Relapse Free Survival | GSE31210 | 218322_s_at |
| **Ovarian** | 278 | 2.88E-02 | 0.82 | Overall Survival | GSE9891 | 222592_s_at |
|  | 278 | 3.76E-02 | 0.84 | Overall Survival | GSE9891 | 218322_s_at |
