# Supplementary material for: Systematic Analysis of Gene Expression Alterations and Clinical Outcomes for Long-Chain Acyl-Coenzyme A Synthetase Family in Cancer
Source: PLoS One. 2016 May 12;11(5):e0155660. doi: 10.1371/journal.pone.0155660 (PMC4865206; doi:10.1371/journal.pone.0155660)
Supplement: S9 Table — (DOC) [file pone.0155660.s012.doc]

| **Supplementary Table 9. ACSL6 expression in cancers** | | | | | | |
| --- | --- | --- | --- | --- | --- | --- |
| **cancer** | cancer subtype | p-value | fold change | rank (%) | sample | Reference |
| **Brain** | Glioblastoma | 9.71E-13 | -4.40 | 4 | 557 | TCGA |
|  | Glioblastoma | 7.29E-14 | -2.74 | 9 | 180 | [1] |
|  |  |  |  |  |  |  |
| **Cervical** | Cervical Squamous Cell Carcinoma | 3.82E-05 | -2.14 | 7 | 45 | [38] |
|  |  |  |  |  |  |  |
| **Colorectal** | Colon Adenoma | 1.10E-07 | 7.50 | 2 | 40 | [18] |
|  | Colon Carcinoma | 1.45E-08 | 19.50 | 4 | 40 | [18] |
|  | Colorectal Adenocarcinoma | 4.42E-14 | 3.34 | 2 | 40 | [18] |
|  | Colorectal Carcinoma | 9.17E-09 | 3.62 | 4 | 40 | [18] |
|  | Colon Adenocarcinoma | 6.19E-24 | 8.58 | 2 | 237 | TCGA |
|  | Rectal Adenocarcinoma | 8.69E-21 | 9.34 | 2 | 237 | TCGA |
|  | Colon Adenocarcinoma | 4.19E-12 | 4.25 | 1 | 105 | [39] |
|  | Rectal Adenocarcinoma | 2.47E-05 | 3.82 | 2 | 105 | [39] |
|  | Cecum Adenocarcinoma | 1.92E-05 | 3.55 | 7 | 105 | [39] |
|  | Rectal Adenocarcinoma | 2.40E-22 | 5.72 | 4 | 130 | [6] |
|  |  |  |  |  |  |  |
| **Head and Neck** | Salivary Gland Adenoid Cystic Carcinoma | 1.54E-04 | -9.11 | 7 | 22 | [9] |
|  |  |  |  |  |  |  |
| **Kidney** | Papillary Renal Cell Carcinoma | 9.35E-22 | -3.24 | 1 | 92 | [40] |
|  | Renal Oncocytoma | 4.62E-20 | -3.66 | 2 | 92 | [40] |
|  | Clear Cell Renal Cell Carcinoma | 3.62E-23 | -3.47 | 2 | 92 | [40] |
|  | Chromophobe Renal Cell Carcinoma | 1.14E-08 | -3.37 | 3 | 92 | [40] |
|  |  |  |  |  |  |  |
| **Leukemia** | Acute Myeloid Leukemia | 4.22E-18 | -2.47 | 5 | 2096 | [41] |
|  | Pro-B Acute Lymphoblastic Leukemia | 1.47E-25 | -3.40 | 6 | 2096 | [41] |
|  | B-Cell Acute Lymphoblastic Leukemia | 1.66E-21 | -2.85 | 8 | 2096 | [41] |
|  | T-Cell Acute Lymphoblastic Leukemia | 5.79E-22 | -2.91 | 8 | 2096 | [41] |
|  | B-Cell Childhood Acute Lymphoblastic Leukemia | 4.55E-22 | -2.93 | 9 | 2096 | [41] |
