# Supplementary material for: Systematic Analysis of Gene Expression Alterations and Clinical Outcomes for Long-Chain Acyl-Coenzyme A Synthetase Family in Cancer
Source: PLoS One. 2016 May 12;11(5):e0155660. doi: 10.1371/journal.pone.0155660 (PMC4865206; doi:10.1371/journal.pone.0155660)
Supplement: S10 Table — (DOC) [file pone.0155660.s013.doc]

| **Supplementary Table 10. The association of ACSL6 expression and the survival in cancer patients** | | | | | | |
| --- | --- | --- | --- | --- | --- | --- |
| **Cancer type** | N | COX P-VALUE | HR | ENDPOINT | DATASET | PROBE ID |
| **Blood** | 163 | 3.53E-02 | 0.73 | Overall Survival | GSE12417-GPL96 | 211207_s_at |
|  | 79 | 9.22E-03 | 0.73 | Overall Survival | GSE12417-GPL570 | 211207_s_at |
|  | 58 | 1.09E-02 | 0.66 | Overall Survival | GSE5122 | 211207_s_at |
|  | 559 | 3.07E-02 | 0.78 | Disease Specific Survival | GSE2658 | 223918_at |
| **Brain** | 74 | 2.69E-02 | 1.4 | Overall Survival | GSE4412-GPL97 | 223918_at |
| **Colorectal** | 226 | 7.72E-03 | 0.81 | Disease Free Survival | GSE14333 | 211207_s_at |
|  | 55 | 4.86E-02 | 0.52 | Overall Survival | GSE17537 | 211207_s_at |
| **Eye** | 63 | 2.54E-06 | 3.33 | Distant Metastasis Free Survival | GSE22138 | 211207_s_at |
| **Lung** | 204 | 2.18E-02 | 2.5 | Overall Survival | GSE31210 | 223918_at |
|  | 204 | 2.10E-02 | 1.86 | Relapse Free Survival | GSE31210 | 223918_at |
