# Supplementary material for: Systematic Analysis of Gene Expression Alterations and Clinical Outcomes for Long-Chain Acyl-Coenzyme A Synthetase Family in Cancer
Source: PLoS One. 2016 May 12;11(5):e0155660. doi: 10.1371/journal.pone.0155660 (PMC4865206; doi:10.1371/journal.pone.0155660)
Supplement: S11 Table — (DOC) [file pone.0155660.s014.doc]

| **Supplementary Table 11. The reference lists in supplementary tables** | |
| --- | --- |
| **1** | Sun L, Hui AM, Su Q, Vortmeyer A, Kotliarov Y, et al. (2006) Neuronal and glioma-derived stem cell factor induces angiogenesis within the brain. Cancer Cell 9: 287-300. |
| **2** | Sorlie T, Perou CM, Tibshirani R, Aas T, Geisler S, et al. (2001) Gene expression patterns of breast carcinomas distinguish tumor subclasses with clinical implications. Proc Natl Acad Sci U S A 98: 10869-10874. |
| **3** | Sorlie T, Tibshirani R, Parker J, Hastie T, Marron JS, et al. (2003) Repeated observation of breast tumor subtypes in independent gene expression data sets. Proc Natl Acad Sci U S A 100: 8418-8423 |
| **4** | Curtis C, Shah SP, Chin SF, Turashvili G, Rueda OM, et al. (2012) The genomic and transcriptomic architecture of 2,000 breast tumours reveals novel subgroups. Nature 486: 346-352. |
| **5** | Scotto L, Narayan G, Nandula SV, Arias-Pulido H, Subramaniyam S, et al. (2008) Identification of copy number gain and overexpressed genes on chromosome arm 20q by an integrative genomic approach in cervical cancer: potential role in progression. Genes, Chromosomes and Cancer 47: 755-765 |
| **6** | Gaedcke J, Grade M, Jung K, Camps J, Jo P, et al. (2010) Mutated KRAS results in overexpression of DUSP4, a MAP-kinase phosphatase, and SMYD3, a histone methyltransferase, in rectal carcinomas. Genes, Chromosomes and Cancer 49: 1024-1034. |
| **7** | Kim SM, Park YY, Park ES, Cho JY, Izzo JG, et al. (2010) Prognostic biomarkers for esophageal adenocarcinoma identified by analysis of tumor transcriptome. PLoS One 5: e15074. |
| **8** | Estilo CL, P Oc, Talbot S, Socci ND, Carlson DL, et al. (2009) Oral tongue cancer gene expression profiling: Identification of novel potential prognosticators by oligonucleotide microarray analysis. BMC Cancer 9: 11. |
| **9** | Frierson HF, Jr., El-Naggar AK, Welsh JB, Sapinoso LM, Su AI, et al. (2002)  Large scale molecular analysis identifies genes with altered expression in  salivary adenoid cystic carcinoma. Am J Pathol 161: 1315-1323. |
| **10** | Andersson A, Ritz C, Lindgren D, Eden P, Lassen C, et al. (2007)  Microarray-based classification of a consecutive series of 121 childhood  acute leukemias: prediction of leukemic and genetic subtype as well as of  minimal residual disease status. Leukemia 21: 1198-1203. |
| **11** | Haferlach C, Bacher U, Schnittger S, Weiss T, Kern W, et al. (2010) Similar patterns of chromosome abnormalities in CML occur in addition to the Philadelphia chromosome with or without tyrosine kinase inhibitor treatment. Leukemia 24: 638-640. |
| **12** | Roessler S, Jia HL, Budhu A, Forgues M, Ye QH, et al. (2010) A unique metastasis gene signature enables prediction of tumor relapse in early-stage hepatocellular carcinoma patients. Cancer Res 70: 10202-10212. |
| **13** | Chen X, Cheung ST, So S, Fan ST, Barry C, et al. (2002) Gene expression patterns in human liver cancers. Mol Biol Cell 13: 1929-1939. |
| **14** | Wurmbach E, Chen YB, Khitrov G, Zhang W, Roayaie S, et al. (2007) Genome-wide molecular profiles of HCV-induced dysplasia and hepatocellular carcinoma. Hepatology 45: 938-947. |
| **15** | Bhattacharjee A, Richards WG, Staunton J, Li C, Monti S, et al. (2001) Classification of human lung carcinomas by mRNA expression profiling reveals distinct adenocarcinoma subclasses. Proc Natl Acad Sci U S A 98: 13790-13795. |
| **16** | Singh D, Febbo PG, Ross K, Jackson DG, Manola J, et al. (2002) Gene  expression correlates of clinical prostate cancer behavior. Cancer Cell 1:  203-209 |
| **17** | Barretina J, Taylor BS, Banerji S, Ramos AH, Lagos-Quintana M, et al. (2010) Subtype-specific genomic alterations define new targets for soft-tissue sarcoma therapy. Nat Genet 42: 715-721 |
| **18** | Skrzypczak M, Goryca K, Rubel T, Paziewska A, Mikula M, et al. (2010)  Modeling oncogenic signaling in colon tumors by multidirectional analyses  of microarray data directed for maximization of analytical reliability. PLoS  One 5. |
| **19** | Talantov D, Mazumder A, Yu JX, Briggs T, Jiang Y, et al. (2005) Novel genes associated with malignant melanoma but not benign melanocytic lesions. Clin Cancer Res 11: 7234-7242 |
| **20** | Bonome T, Levine DA, Shih J, Randonovich M, Pise-Masison CA, et al. (2008) A gene signature predicting for survival in suboptimally debulked patients with ovarian cancer. Cancer Res 68: 5478-5486 |
| **21** | Sanchez-Carbayo M, Socci ND, Lozano J, Saint F, Cordon-Cardo C (2006) Defining molecular profiles of poor outcome in patients with invasive bladder cancer using oligonucleotide microarrays. J Clin Oncol 24: 778-789 |
| **22** | Bredel M, Bredel C, Juric D, Harsh GR, Vogel H, et al. (2005) High-resolution genome-wide mapping of genetic alterations in human glial brain tumors. Cancer Res 65: 4088-4096 |
| **23** | Finak G, Bertos N, Pepin F, Sadekova S, Souleimanova M, et al. (2008) Stromal gene expression predicts clinical outcome in breast cancer. Nature Medicine 14: 518-527 |
| **24** | Ginos MA, Page GP, Michalowicz BS, Patel KJ, Volker SE, et al. (2004) Identification of a gene expression signature associated with recurrent disease in squamous cell carcinoma of the head and neck. Cancer Res 64: 55-63 |
| **25** | Peng CH, Liao CT, Peng SC, Chen YJ, Cheng AJ, et al. (2011) A novel molecular signature identified by systems genetics approach predicts prognosis in oral squamous cell carcinoma. PLoS One 6: e23452 |
| **26** | Jones J, Otu H, Spentzos D, Kolia S, Inan M, et al. (2005) Gene signatures of progression and metastasis in renal cell cancer. Clin Cancer Res 11: 5730-5739 |
| **27** | Mas VR, Maluf DG, Archer KJ, Yanek K, Kong X, et al. (2009) Genes involved in viral carcinogenesis and tumor initiation in hepatitis C virus-induced hepatocellular carcinoma. Mol Med 15: 85-94 |
| **28** | Hou J, Aerts J, den Hamer B, van Ijcken W, den Bakker M, et al. (2010) Gene expression-based classification of non-small cell lung carcinomas and survival prediction. PLoS One 5: e10312 |
| **29** | Selamat SA, Chung BS, Girard L, Zhang W, Zhang Y, et al. (2012) Genome-scale analysis of DNA methylation in lung adenocarcinoma and integration with mRNA expression. Genome Res 22: 1197-1211 |
| **30** | Wei TY, Juan CC, Hisa JY, Su LJ, Lee YC, et al. (2012) Protein arginine methyltransferase 5 is a potential oncoprotein that upregulates G1 cyclins/cyclin-dependent kinases and the phosphoinositide 3-kinase/AKT signaling cascade. Cancer Sci 103: 1640-1650 |
| **31** | Zhan F, Barlogie B, Arzoumanian V, Huang Y, Williams DR, et al. (2007) Gene-expression signature of benign monoclonal gammopathy evident in multiple myeloma is linked to good prognosis. Blood 109: 1692-1700 |
| **32** | Buchholz M, Braun M, Heidenblut A, Kestler HA, Kloppel G, et al. (2005) Transcriptome analysis of microdissected pancreatic intraepithelial neoplastic lesions. Oncogene 24: 6626-6636 |
| **33** | Badea L, Herlea V, Dima SO, Dumitrascu T, Popescu I (2008) Combined gene expression analysis of whole-tissue and microdissected pancreatic ductal adenocarcinoma identifies genes specifically overexpressed in tumor epithelia. Hepatogastroenterology 55: 2016-2027 |
| **34** | Richardson AL, Wang ZC, De Nicolo A, Lu X, Brown M, et al. (2006) X chromosomal abnormalities in basal-like human breast cancer. Cancer Cell 9: 121-132 |
| **35** | Okayama H, Kohno T, Ishii Y, Shimada Y, Shiraishi K, et al. (2012) Identification of genes upregulated in ALK-positive and EGFR/KRAS/ALK-negative lung adenocarcinomas. Cancer Res 72: 100-111. |
| **36** | Iacobuzio-Donahue CA, Maitra A, Olsen M, Lowe AW, van Heek NT, et al. (2003) Exploration of global gene expression patterns in pancreatic adenocarcinoma using cDNA microarrays. Am J Pathol 162: 1151-1162 |
| **37** | Grasso CS, Wu YM, Robinson DR, Cao X, Dhanasekaran SM, et al. (2012) The mutational landscape of lethal castration-resistant prostate cancer. Nature 487: 239-243 |
| **38** | Biewenga P, Buist MR, Moerland PD, Ver Loren van Themaat E, van Kampen AH, et al. (2008) Gene expression in early stage cervical cancer. Gynecologic Oncology 108: 520-526 |
| **39** | Kaiser S, Park YK, Franklin JL, Halberg RB, Yu M, et al. (2007) Transcriptional recapitulation and subversion of embryonic colon development by mouse colon tumor models and human colon cancer. Genome Biology 8: R131 |
| **40** | Jones TD, Eble JN, Wang M, MacLennan GT, Delahunt B, et al. (2005) Molecular genetic evidence for the independent origin of multifocal papillary tumors in patients with papillary renal cell carcinomas. Clin Cancer Res 11: 7226-7233 |
| **41** | Haferlach T, Kohlmann A, Wieczorek L, Basso G, Kronnie GT, et al. (2010) Clinical utility of microarray-based gene expression profiling in the diagnosis and subclassification of leukemia: report from the International Microarray Innovations in Leukemia Study Group. J Clin Oncol 28: 2529-2537 |
